# MON2 Guides Wntless Transport to the Golgi through Recycling Endosomes

**DOI:** 10.1247/csf.20012

**Published:** 2020-05-12

**Authors:** Shen-Bao Zhao, Neta Dean, Xiao-Dong Gao, Morihisa Fujita

**Affiliations:** 1 Key Laboratory of Carbohydrate Chemistry, Ministry of Education, School of Biotechnology, Jiangnan University, Wuxi, Jiangsu 214122, China; 2 Department of Biochemistry and Cell Biology, Stony Brook University, Stony Brook, NY, 11794-5215, USA

**Keywords:** membrane trafficking, MON2, recycling endosomes, Wntless

## Abstract

Endocytic cargos are transported to recycling endosomes (RE) but how these sorting platforms are generated is not well understood. Here we describe our biochemical and live imaging studies of the conserved MON2-DOPEY complex in RE formation. MON2 mainly co-localized with RE marker RAB4B in peripheral dots and perinuclear region. The peripheral RE approached, interacted with, and separated from sorting nexin 3 (SNX3)-positive early endosomes (EE). Membrane-bound DOPEY2 was recruited to RE dependent upon MON2 expression, and showed binding abilities to kinesin and dynein/dynactin motor proteins. MON2-knockout impaired segregation of RE from EE and led to a decreased tubular recycling endosomal network, whereas RE was accumulated at perinuclear regions in DOPEY2-knockout cells. MON2 depletion also impaired intracellular transferrin receptor recycling, as well as retrograde transport of Wntless during its passage through RE before delivery from EE to the Golgi. Together, these data suggest that the MON2 drives separation of RE from EE and is required for efficient transport of endocytic cargo molecules.

## Introduction

A balance between synthesis and degradation of proteins is required to maintain the homeostasis of cellular processes (Cullen and Steinberg, 2018). The endosomal system contributes to this balance through vesicular sorting of proteins for degradation and recycling to various membrane compartments. Mammalian cells contain a complex set of endosomal compartments, including early endosomes (EE), late endosomes (LE), recycling endosomes (RE), and lysosomes (Klumperman and Raposo, 2014). The EE are the first carrier vesicles into which plasma membrane (PM) proteins and lipids enter when internalized (Jovic *et al.*, 2010). EE undergo structural and functional maturation to form either LE (Scott *et al.*, 2014), whose cargos are transported to lysosomes for degradation or RE, whose cargos are sorted and transported back to the PM, or the Golgi (Taguchi, 2013). Although RE play pivotal roles in maintaining cellular homeostasis, the mechanism of how they regulate protein and lipid sorting is not well understood.

RE are mainly localized at the perinuclear regions as transient vesicular/tubular structures as well as peripheral dot-like puncta (Grant and Donaldson, 2009; Maxfield and McGraw, 2004). The formation and maintenance of endosomal compartments involve a host of different Rab GTPases (Pfeffer, 2013, 2017) whose GTP-bound state at membranes is required for vesicle formation from the donor or vesicle fusion with the acceptor. RAB4 is found at tubular structures of EE or RE (D’Souza *et al.*, 2014; de Wit *et al.*, 2001; Perrin *et al.*, 2013; Sonnichsen *et al.*, 2000), while RAB11 is mainly enriched in RE (Green *et al.*, 1997; Ullrich *et al.*, 1996). To date, several multiprotein complexes have been reported that cooperate to generate and move tubules from endosomes (Gautreau *et al.*, 2014). Rab GTPases and kinesin provide the mechanical force for tubularization and fission of tubular/vesicular structures from the EE membrane (Delevoye *et al.*, 2014; Etoh and Fukuda, 2019; Shakya *et al.*, 2018). Most kinesins mediate transport toward the plus-ends of microtubules (Hirokawa *et al.*, 2009). However, RE are clustered around the microtubule-organizing center (MTOC) (Maxfield and McGraw, 2004) located at the minus-ends of microtubules (Martin and Akhmanova, 2018). Thus, it is likely that coordination of other factors and motor proteins regulates RE tubular network and movement.

Yeast Mon2 is a peripheral membrane protein in the *trans*-Golgi network (TGN) (Efe *et al.*, 2005) that, with its cytosolic binding partner, Dop1, is implicated in endosome-to-Golgi transport (Gillingham *et al.*, 2006). We recently described a role for yeast Dop1 in retrograde transport from the TGN to the Golgi (Zhao *et al.*, 2019). The yeast TGN is assumed to be functionally equivalent to mammalian EE and RE (Day *et al.*, 2018). Mon2 and Dop1 has been highly conserved through evolution. In metazoans, there are two Dop1 orthologues, named DOPEY1 and DOPEY2 (Barbosa *et al.*, 2010; Mahajan *et al.*, 2019; McGough *et al.*, 2018). Even DOPEY1 is monomer and DOPEY2 is self-interacted, both DOPEY1 and DOPEY2 were reported to interact with mammalian MON2 (Mahajan *et al.*, 2019; McGough *et al.*, 2018). DOPEY1 interacts with the KLC2, the light chain of kinesin-1 motor protein (Mahajan *et al.*, 2019), suggesting that MON2-DOPEY complex mediates vesicular/tubular movement.

Retrograde transport of Wntless (Wls), which is a critical cargo receptor for Wnt secretion (Banziger *et al.*, 2006), from EE to the Golgi requires mammalian MON2-DOPEY2 (McGough *et al.*, 2018). After internalization of Wls from the PM, it is transported to EE, and then transported to the Golgi (Harterink *et al.*, 2011; Port *et al.*, 2008). In *Caenorhabditis elegans*, Wls is transported from RE to the Golgi (Bai and Grant, 2015). However, it remains unclear whether Wls is directly transported from EE to the Golgi or through RE in mammalian cells. It is because RE are mainly localized at the perinuclear Golgi regions as transient vesicular/tubular structures as well as peripheral dot-like puncta (Grant and Donaldson, 2009; Maxfield and McGraw, 2004). Recent findings suggest that both the TGN and RE are functionally overlapping and RE attach to the trans-side of Golgi stacks under nocodazole treatment (Fujii *et al.*, 2020). It is still not clear how MON2-DOPEY proteins are involved in Wls retrograde trafficking.

Here, we show that the MON2 mainly localizes at RE in human embryonic kidney 293 (HEK293) cells. Membrane-bound DOPEY proteins were recruited to RE dependent upon MON2 expression. *MON2* knockout (MON2-KO) impaired the segregation of RE from EE. Wls passed through RE dependent upon MON2 for transport to the Golgi. These findings suggest that the MON2-DOPEY complex regulates cargo transport by regulating RE movement.

## Material and Methods

### Cell culture and gene editing by CRISPR/Cas9

HEK293 cells were cultured in Dulbecco’s modified Eagle’s medium (DMEM) supplemented with 10% fetal bovine serum. Cells stably expressing 3×FLAG-tagged DOPEY1 or DOPEY2 were obtained by transfection of pME-Hyg-DOPEY1-3FLAG or pME-Hyg-DOPEY2-3FLAG and selection with hygromycin B (400 μg/ml). Transfection of DNA constructs was performed using Lipofectamine 2000 (Thermo Fisher, Waltham, MA) according to the manufacturer’s instructions. To generate *MON2*, *DOPEY1*, *DOPEY2* and *VPS35* gene KO cell lines, HEK293 cells were transiently transfected with pX330-EGFP plasmids containing the target sequences. After 48 h, cells with EGFP were sorted by an S3e Cell Sorter (Bio-Rad Laboratories, Hercules, CA). The collected cells were cultured for more than 10 days and subjected to limiting dilution to obtain clonal KO cells. Genomic DNA was extracted from individual clones and subjected to PCR. Clones with no wild-type (WT) allele were selected, and the DNA sequences around the KO targets were analyzed. The DNA sequences of the individual KO cell lines are listed in Supplemental Table S1.

### Antibodies and reagents

The following commercial antibodies were used: mouse anti-FLAG (#F3165; Sigma-Aldrich, St. Louis, MO), rabbit anti-FLAG (#20543-1-AP; Proteintech, Chicago, IL), rabbit anti-HA (#3724; Cell Signaling Technology, Danvers, MA), mouse anti-HA (#H3663; HA-7; Sigma-Aldrich), mouse anti-Golgin97 (#97537; Cell Signaling Technology), rabbit anti-Golgin97 (#13192; Cell Signaling Technology), mouse anti-GM130 (#610823; BD Transduction Laboratories, Franklin Lakes, NJ), mouse anti-β-tubulin (#HC101-01; TransGen Biotech, Beijing, China), mouse anti-LAMP1 (H4A3; Developmental Studies Hybridoma Bank, Iowa City, IA), rabbit anti-VPS35 (#ab157220; Abcam), rabbit anti-calnexin (#C4731; Sigma-Aldrich), mouse anti-Giantin (#ab37266; Abcam), rabbit anti-DOPEY1 (#HPA027902; Sigma-Aldrich), rabbit anti-MON2 (#ab206685; Abcam), rabbit anti-tRFP (#AB233; Evrogen, Moscow, Russia), mouse anti-TfnR (#13-6800; Thermo Fisher), rabbit anti-SNX3 (#10772-1-AP; Proteintech). Human Transferrin CF^TM^488A (#00081; Biotium, Fremont, CA). Nocodazole (#M1404; Sigma-Aldrich). Anti-rabbit or anti-mouse IgG Alexa Fluor-conjugated secondary antibodies (Thermo Fisher) were used for detection by confocal microscopy. HRP-conjugated anti-mouse IgG (#HS211-01; TransGen Biotech) and anti-rabbit IgG (#HS101-01; TransGen Biotech) were used as secondary antibodies for western blotting.

### Plasmids

The plasmids for target gene KO were constructed as previously described (Liu *et al.*, 2018). Human genes were amplified from cDNAs isolated from HEK293 or HeLa cells. Alternatively, cDNAs from Ultimate^TM^ ORF LITE clones (Thermo Fisher) were used as templates. In-fusion PCR cloning or restriction enzyme digestion followed by ligation was used for plasmid construction. Full-length DOPEY1 was ligated into the XhoI/MluI site of pME-Hyg-3FLAG to generate pME-Hyg-DOPEY1-3FLAG. pME-Hyg-DOPEY2-3FLAG was obtained by in-fusion cloning of full-length DOPEY2 into the EcoRI/MluI site of pME-Hyg-3FLAG. pME-Hyg-DOPEY1-FLAG-EGFP and pME-Hyg-DOPEY2-FLAG-EGFP were obtained by replacement of the 3FLAG fragment in pME-Hyg-DOPEY1-3FLAG or DOPEY2-3FLAG with the FLAG-EGFP fragment, respectively.

The plasmid pME-3HA-MON2 was obtained by in-fusion cloning of full-length MON2 into the MluI/NotI site of pME-3HA. pME-EGFP-MON2 and pME-TagRFP-MON2 were obtained by replacement of the 3HA fragment in pME-3HA-MON2 with EGFP or TagRFP, respectively. pME-EGFP-RAB4A, pME-EGFP-RAB4B, pME-EGFP-RAB5, pME-EGFP-RAB7, and pME-EGFP-RAB11 were obtained by in-fusion cloning of each full-length gene into the SalI/NotI site of pME-EGFP. pME-EGFP-RAB5Q79L was obtained by site-directed mutagenesis of pME-EGFP-RAB5. pME-TagRFP-RAB4B was obtained by in-fusion cloning of full-length RAB4B into the SalI/NotI site of pME-TagRFP. pME-BFP-SNX3 and pME-EGFP-SNX3 were obtained by in-fusion cloning of full-length SNX3 into the SalI/XbaI site of pME-BFP and pME-EGFP, respectively.

The plasmid pME-Wls-EGFP was obtained by in-fusion cloning of full-length Wls into the EcoRI/MluI site of pME-EGFP. To construct pME-Hyg-HA-Wls as described previously (Varandas *et al.*, 2016), we inserted the HA fragment into the largest extracellular loop and C-terminus of Wls. The plasmid mScarlet-Giantin was obtained from Addgene (#85048). The plasmid mNeonGreen-Giantin was obtained from Addgene (#98880). pME-KLC2-3HA was obtained by in-fusion cloning of full-length KLC2 into the XhoI/MluI site of pME-3HA. pME-3HA-DCTN1 was obtained by in-fusion cloning of full-length DCTN1 into the MluI/NotI site of pME-3HA. pME-sOGT-3HA was obtained by in-fusion cloning of full-length sOGT into the MluI/NotI site of pME-3HA.

### Confocal microscopy

For fixed samples, glass-bottom dishes (#D35-10-1-N; Cellvis, Mountain View, CA) or sterile glass coverslips in culture dishes were coated with 0.1% gelatin (#G7041; Sigma-Aldrich) for 15 min at room temperature, followed by complete removal of excess gelatin. After transfection, cells were seeded into 24-well plates with sterile glass coverslips or into glass-bottom dishes. When the cell density reached about 60% confluence, the cells were fixed with –20°C cold methanol as described previously (Bhattacharyya *et al.*, 2010). Alternatively, the cells were fixed with 4% paraformaldehyde solution (#P0099; Beyotime, Shanghai, China) at room temperature for 20 min, permeabilized with Saponin (#P0095; Beyotime) for 10 min, and blocked with blocking buffer (#P0260; Beyotime) for 1 h at room temperature. The cells were then incubated with primary antibodies diluted in QuickBlock^TM^ Primary Antibody Dilution Buffer (#P0262; Beyotime) overnight at 4°C. After five gentle washes with phosphate-buffered saline (PBS), the cells were incubated with an Alexa Fluor-conjugated secondary antibody (Thermo Fisher) at room temperature for 1 h. Finally, the cells were washed gently eight times with PBS and mounted on slides in Antifade Mounting Medium (#P0126; Beyotime) with or without Hoechst 33258 dye. Images were obtained with a Nikon Eclipse Ti-E inverted confocal microscope equipped with a Plan Apo 100×/1.4 NA oil objective and a C2si scan head camera. NIS-Element AR software (Nikon, Tokyo, Japan) was used to operate the microscope. MCCs were calculated using the JACoP plugin of ImageJ (https://imagej.nih.gov/ij/). We first set the appropriate threshold value of the pixels for both color channels (e.g., Red vs Green). The M1 coefficient represented the ratio of the “sum of pixels from the former channel (Red) that colocalizes with the latter channel (Green)” to the “total intensity of pixels in the former channel (Red)”. M2 measured the opposite channel to M1.

### Live-cell microscopy

Cells were cultured and replated onto glass-bottom dishes precoated with 0.1% gelatin. When the cell density reached about 60% confluence, the cells were imaged in pre-warmed FluoroBrite^TM^ DMEM (#A1896701; Thermo Fisher) supplemented with 10% fetal bovine serum. Images were obtained with a Leica SP8 (Wetzlar, Germany) scanning confocal microscope equipped with a Plan Apo 60×/1.4 NA oil objective and solid-state lasers. We set the frame size to 512×512 pixels, the digital zoom to 1.5–2.5 for one or two cells, and the pinhole to 1.8 Airy units with bidirectional mode for scanning. The highest sensitivity detector was used for the weaker fluorescence of TagRFP-MON2. All live image videos can be downloaded from: https://drive.google.com/drive/folders/1beD9ONP9k8yKECHoApZhTgk1qomEHxlz?usp=sharing

### Wls internalization assay

For internalization of surface-labeled HA-Wls, transfected cells were replated into 24-well plates, rinsed with ice-cold PBS, and incubated with a rabbit anti-HA antibody (#3724; Cell Signaling Technology) diluted 1:250 in OPTI-MEM (#31985062; Thermo Fisher) for 15 min. After a gentle wash with PBS to remove unbound antibody, an internalization assay was performed by placing the cells in normal medium at 37°C. The cells were then fixed with –20°C cold methanol, incubated with a DyLight 405-labeled goat anti-rabbit secondary antibody (#A0605; Beyotime), and imaged.

### Cell fractionation

Cells stably expressing DOPEY1-3FLAG or DOPEY2-3FLAG (1×10^7^) were placed in 1 ml of hypotonic buffer (10 mM Tris-HCl, pH 7.4, 1 mM Dithiothreitol, 0.2 mM MgCl_2_, 5 mM KCl, protein inhibitor cocktail, 1 mM PMSF), allowed to swell on ice for 5 min, and lysed by 20 strokes in a Dounce homogenizer. The nuclei were removed from the homogenate by centrifugation at 1000×*g* for 15 min at 4°C. Sucrose was added to the supernatant to a final concentration of 0.25 M, followed by centrifugation at 10,000×*g* for 1 h at 4°C. The resulting pellet (P10) containing the PM and endoplasmic reticulum membrane was resuspended in 1 ml of lysis buffer (50 mM Tris-HCl, pH 7.5, 100 mM NaCl, 1 mM EDTA, protease inhibitor cocktail, 1 mM PMSF, 1% NP-40), and centrifuged at 100,000×*g* for 1 h at 4°C. The obtained pellet (P100) containing the Golgi, lysosomes, endosomes, and other membrane vesicles was resuspended in 1 ml of lysis buffer. Finally, the supernatant (S100) containing cytoplasmic proteins was adjusted to 1 ml with lysis buffer. Tubulin was used as the control for the fractionation.

### Immunoprecipitation

Cells were lysed in lysis buffer (50 mM Tris-HCl, pH 7.5, 100 mM NaCl, 1 mM EDTA, protease inhibitor cocktail, 1 mM PMSF, 1% NP-40) on ice for 30 min, and centrifuged at 21,500×*g* for 10 min to remove any intact cells and cell debris. Anti-FLAG M2 affinity gel (#A2220; Sigma-Aldrich) was added to the supernatant and rotated at 4°C for 1 h. After four gentle washes with PBS, the bound proteins were eluted by FLAG peptide or direct boiling in SDS sample buffer. Samples were analyzed by immunoblotting or silver staining. The bands corresponding to DOPEY2 and MON2 were identified by liquid chromatography-tandem mass spectrometry in which performed by Shanghai Applied Protein Technology Co.,Ltd (Shanghai, China).

### SDS-PAGE and immunoblotting

Samples were separated in 8% or 4%–20% SDS-PAGE gels, and transferred to PVDF membranes. The membranes were blocked in 5% milk in TBST buffer (10 mM Tris-HCl, pH 7.5, 150 mM NaCl, 0.05% (v/v) Tween-20) and probed with appropriate antibodies diluted in QuickBlock^TM^ Primary Antibody Dilution Buffer (#P0256; Beyotime) for 1 h at room temperature or 4°C overnight. After three washes with TBST for 10 min each, the membranes were incubated with HRP-conjugated secondary antibodies diluted in 5% milk in TBST buffer for 1 h at room temperature, and washed three times in TBST buffer. Bound antibodies were detected with ECL Substrate (#1705060; Bio-Rad). For reuse of the membranes to detect the loading control, the membranes were treated with stripping buffer (#P0025N; Beyotime). Images were captured using an Amersham Imager 680 Camera System (GE Healthcare, Princeton, NJ) or Tanon 5200 Automatic Chemiluminescence Image Analysis System (Shanghai, China). The band intensities were quantified using ImageJ.

### Transferrin recycling assay

HEK293 WT or MON2-KO cells were incubated with CF^TM^488A -coupled Tfn (40 μg/ml) at 4°C for 1 h, followed by rapid washing with acid wash buffer (50 mM glycine, 100 mM NaCl, pH 3.0) for 2 min. The cells were then incubated with prewarmed medium in the presence of excess Holo-Tfn (100 μg/ml) for specified periods of time at 37°C. At the end of the incubation, the cells were gently washed with PBS and fixed with cold methanol for confocal analysis. The amounts of intracellular Tfn in six fields were quantified by ImageJ and normalized by the cell number in each field.

## Results

### Both DOPEY1 and DOPEY2 interact with MON2

**Fig. 1:**
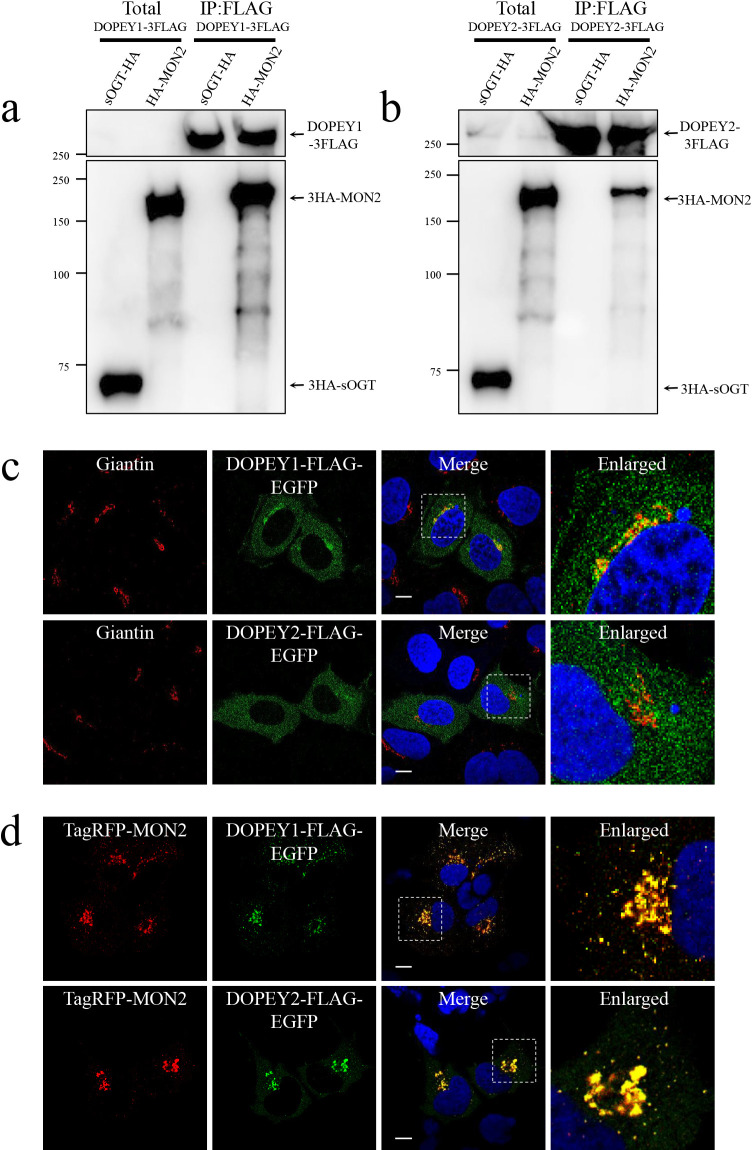
MON2 associates with both DOPEY1 and DOPEY2. a and b, HEK293 cells stably expressing DOPEY1-3FLAG (a) or DOPEY2-3FLAG (b) were transiently transfected with 3HA-sOGT or 3HA-MON2. Cell lysates were incubated with anti-FLAG beads, and precipitates were immunoblotted with the indicated antibodies. 3HA-sOGT was used as a negative control. c and d, Representative confocal images of EGFP-tagged DOPEY1 (c) and DOPEY2 (d). HEK293 cells were transiently transfected with the indicated constructs. Endogenous Giantin was immunostained as a Golgi marker. The enlarged images show magnified views of the boxed areas. Scale bars, 10 μm.

To better understand DOPEY functions, we searched for DOPEY2 interacting proteins. A ~200 kDa protein that specifically immunoprecipitated with 3×FLAG-tagged DOPEY2 (DOPEY2-3FLAG) was identified by mass spectrometry as MON2 (Supplemental Figure S1a). Recently, McGough *et al.* showed that DOPEY2 associates with MON2 (McGough *et al.*, 2018), while Mahajan *et al.* reported that DOPEY1, but not DOPEY2, binds to MON2 (Mahajan *et al.*, 2019). We confirmed that MON2 immunoprecipitated with both DOPEY1-3FLAG or DOPEY2-3FLAG (Fig. 1a, b).

To probe their interaction, we analyzed the localization of DOPEY1, DOPEY2 and MON2. Subcellular fractionation revealed that, as expected, DOPEY1 and DOPEY2 were mainly associated with membranes (Supplemental Figure S1b). GFP-tagged DOPEY1 (DOPEY1-FLAG-EGFP) showed weak co-localization with a Golgi marker, Giantin, with the majority of DOPEY1 localized in punctate foci throughout the cytoplasm (Fig. 1c). In contrast to DOPEY1, DOPEY2 was not detectable at all in the Golgi and was only present in these cytoplasmic foci (Fig. 1c). Because both DOPEY1 and DOPEY2 interacted with MON2 in mammalian cells, we monitored DOPEY1-FLAG-EGFP and DOPEY2-FLAG-EGFP localization as a function of MON2 expression (TagRFP-MON2). As shown in Fig. 1d, both DOPEY1 and DOPEY2 were strongly recruited to perinuclear structures after co-expression with TagRFP-MON2. These results indicate that MON2 could associate with both DOPEY1 and 2 proteins on the membranes.

### Mammalian MON2 mainly localizes at RE

**Fig. 2:**
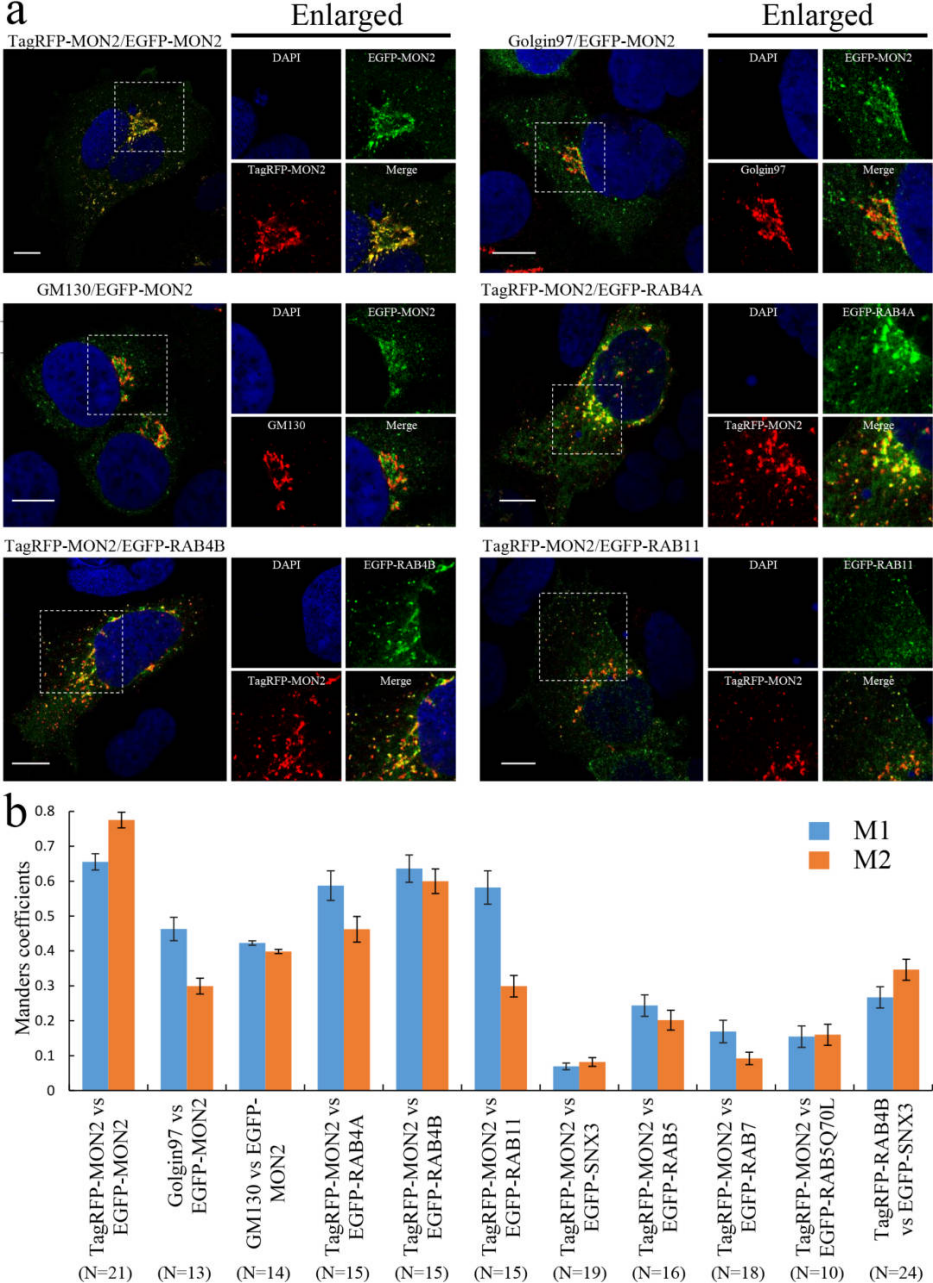
Subcellular localization of MON2. a, Representative confocal images of MON2. HEK293 cells were transiently transfected with constructs for expression of fluorescent-tagged proteins. Endogenous Golgin97 and GM130 were immunostained as Golgi markers. Enlarged images show magnified views of the boxed areas. Scale bars, 10 μm. b, Quantitative analysis of protein co-localization. MCCs between two proteins were calculated using the ImageJ plugin JACoP. M1 represents the fraction of the red channel that overlapped with the green channel. M2 represents the fraction of the green channel that overlapped with the red channel. The data represent means±SE of the measurements. The numbers of cells used in the calculations are indicated in the parentheses.

Since MON2 has been implicated as scaffolding protein that recruits cytosolic DOPEY proteins to the specific membrane structures, we next determined MON2 localization in HEK293 cells. GFP-tagged MON2 (EGFP-MON2) almost completely co-localized with TagRFP-tagged MON2 (TagRFP-MON2), suggesting that MON2 localization is the same whether it is tagged with GFP or RFP (Fig. 2a). Perinuclear MON2 co-localized with the trans-Golgi/TGN marker Golgin97 and cis-Golgi marker GM130 (Fig. 2a). Since endocytic recycling compartments are concentrated between the Golgi ribbon (Saraste and Prydz, 2019) and recent findings revealed that RE attach to the trans-side of Golgi stacks (Fujii *et al.*, 2020), we sought to determine if MON2 is enriched in endosomes rather than the Golgi. To verify where MON2 positions in the Golgi and endosomes, we treated cells with nocodazole to introduce mini-Golgi stacks. After 3 h nocodazole treatment, TagRFP-MON2 was localized to trans-side of mini-Golgi stacks by comparison with GM130 (cis-Golgi)/Giantin (medial-Golgi) or Giantin /Golgin97 (trans-Golgi/TGN) (Supplemental Figure S2a, b). In addition, MON2 was separated from Golgin97, suggesting that MON2 is not localized in the trans-Golgi/TGN (Supplemental Figure S2b). To further determine the MON2 localization, we compared MON2 to various endosomal markers, including those for EE, LE, and RE. The small Rab GTPases RAB4A and RAB4B are enriched at the tubular subdomain of EE and RE (D’Souza *et al.*, 2014; Perrin *et al.*, 2013), whereas RAB11 is localized to RE (Green *et al.*, 1997; Ren *et al.*, 1998; Ullrich *et al.*, 1996). The degree with which MON2 and each of these markers co-localized was quantified by Manders correlation coefficients (MCCs), which measures the proportionality of pixel intensity (Bolte and Cordelieres, 2006) (Fig. 2b). These analyses demonstrated TagRFP-MON2 predominantly co-localized with RAB4A, RAB4B, or RAB11 in the RE (Fig. 2a). MON2 co-localized predominantly with RAB4B: M1 of MCC=0.636±0.039 (TagRFP-MON2 overlapping with EGFP-RAB4B) and M2 of MCC=0.599±0.035 (EGFP-RAB4B overlapping with TagRFP-MON2) (n=15 for both, mean±SE), meaning that 63.6% of MON2 overlapped with RAB4B and 59.9% of RAB4B overlapped with MON2. RAB4A and RAB11 also co-localized with MON2, while the M2 values (the percentage of EGFP-RAB4A or RAB11 overlapping with TagRFP-MON2) were slightly lower.

We also examined MON2 co-localization with sorting nexin SNX3 as it was reported to interact with MON2 during endosome vesicle/tubule formation (McGough *et al.*, 2018). SNX3 is an EE marker; it specifically binds to phosphatidylinositol-3-phosphate on the membrane of EE and mediates SNX3-retromer complex-dependent vesicular/tubular structure formation (Harterink *et al.*, 2011; McGough *et al.*, 2018; Xu *et al.*, 2001). In comparisons of TagRFP-MON2 with EGFP-SNX3, the M1 of MCC was 0.069±0.009 and the M2 of MCC was 0.082±0.012 (n=19 for both, mean±SE), suggesting MON2 and SNX3 rarely co-localized (Fig. 2b and Supplemental Figure S3). A similar lack of co-localization was also observed for other EE and LE markers such as RAB5 and RAB7 (Fig. 2b and Supplemental Figure S3). Taken together, our results indicate that MON2 is primarily localized to RE.

### RAB4B-positive RE transiently interact with EE

**Fig. 3:**
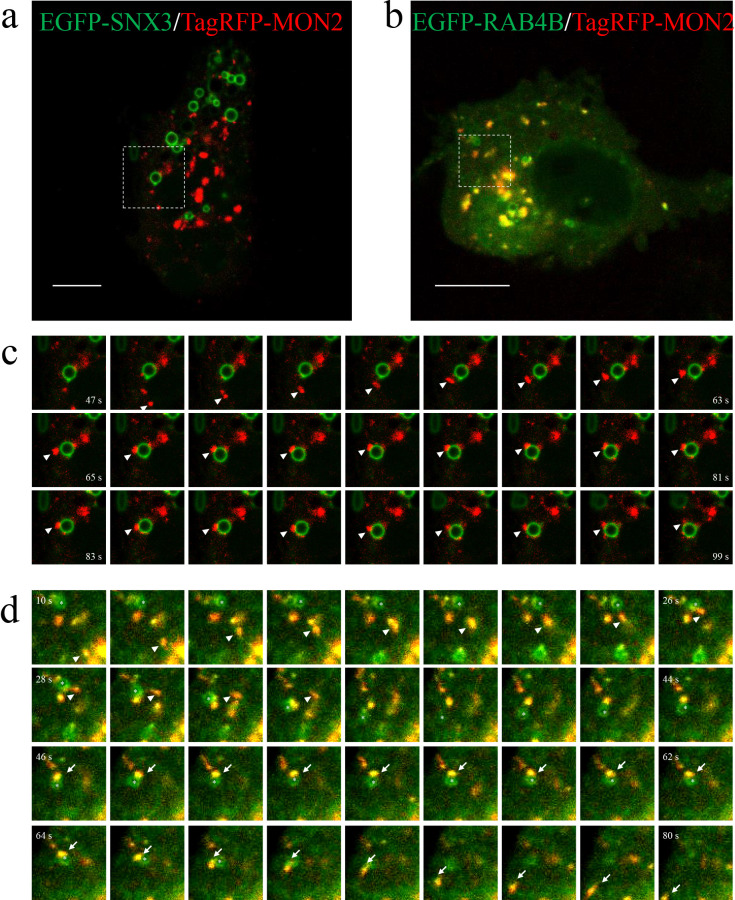
Transient contact between the MON2-positive compartment and an SNX3-labeled EE. a and b, Representative images from time-lapse videos (Movies 1 and 2, https://drive.google.com/drive/folders/1beD9ONP9k8yKECHoApZhTgk1qomEHxlz?usp=sharing) of HEK293 cells co-expressing TagRFP-MON2 and EGFP-SNX3 (a) or EGFP-RAB4B (b). Scale bars, 10 μm. c, Time-lapse images of the area shown in (a) at the indicated time points. The arrowheads indicate a representative TagRFP-MON2-labeled structure approaching and making contact with an EGFP-SNX3-labeled endosome. d, Time-lapse images of the area shown in (b) at the indicated time points. The arrowheads and arrows indicate movement of a compartment labeled by TagRFP-MON2 and EGFP-RAB4B that approached, made contact with, and separated from a ring-shaped endosome marked by the asterisk.

To examine the dynamics and interactions of MON2 with endosomes in living cells, we co-expressed EGFP-SNX3 or EGFP-RAB4B with TagRFP-MON2 in HEK293 cells and imaged the cells by confocal microscopy (Fig. 3a, b). EEs form ring-like structures in some cell types (Wilson *et al.*, 2000). Similar to RAB5 (Hirota *et al.*, 2007), EEs showed larger ring-like shapes in some cells when EGFP-SNX3 was overexpressed. These SNX3-EE rings advantageously enabled a clearer observation of MON2 contacts with endosomal membranes (Fig. 3c and Movie 1, all movies can be downloaded from: https://drive.google.com/drive/folders/1beD9ONP9k8yKECHoApZhTgk1qomEHxlz?usp=sharing). Time-lapse imaging demonstrated MON2-positive RE often approached and transiently contacted the edge of SNX3-positive EE ring. SNX3-positive EE that associated with MON2-positive RE did not fuse; rather, these EE remained segregated and subsequently retreated back to the cytoplasm. RAB4B co-localized with MON2-positive RE and displayed the same bidirectional movement. Notably, this movement was observed in the vesicular/tubular structures (marked by arrowheads in Fig. 3d) that approached, made contact with, and separated from the ring-shaped endosomes (Movie 2). These results indicate that RE transiently interact with EE through bidirectional movement.

### MON2 is required for RE segregation from EE

**Fig. 4:**
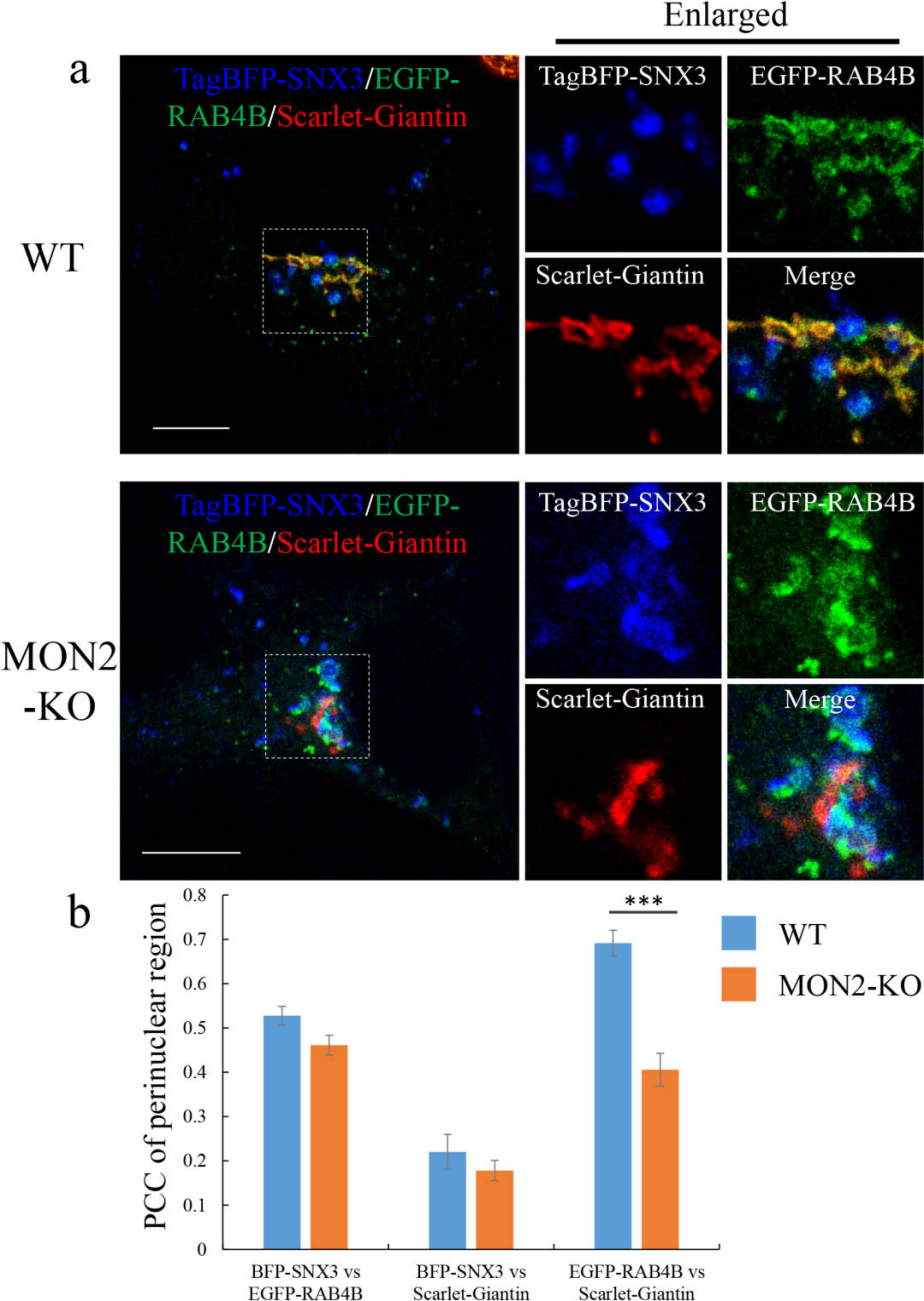
Localization of RE in WT and MON2-KO cells. a, Representative merged confocal images of RAB4B-positive RE. HEK293 WT or MON2-KO cells were transiently transfected with TagBFP-SNX3, EGFP-RAB4B, and Scarlet-Giantin. Enlarged images show magnified views of the boxed areas. Scale bars, 10 μm. b, Quantitative analysis of co-localization between TagBFP-SNX3, EGFP-RAB4B, and Scarlet-Giantin in the perinuclear region. PCCs between two proteins were calculated using the ImageJ plugin JACoP. The data represent means±SE of the measurements (N=12 for WT cells; N=10 for MON2-KO cells). ***, *P*<0.001 (two-tailed Student’s *t* test).

To examine MON2 function on RE trafficking, we examined loss of MON2 or DOPEY function on localization of RE labeled by RAB4B. EE and Golgi were marked by TagBFP-SNX3 and Scarlet-Giantin, respectively. All gene KO in this study were confirmed by genome sequence (Supplemental Table 1) and immunoblotting with available antibodies against MON2, VPS35, and DOPEY1 (Supplemental Figure S4a). In WT cells, RAB4B-positive RE localized in three places: the perinuclear region near the Golgi ribbon, the tubular/vesicular structures associated with the EE membrane, and the peripheral cytoplasmic dots (Fig. 4a). In contrast, RAB4B fluorescence in the Giantin-positive Golgi was almost undetectable in cells deleted for MON2. Instead, in MON2-KO cells, RAB4B co-localized with SNX3, suggesting it was mainly in EE region (Fig. 4a). Quantification of RAB4B co-localization with these markers in the perinuclear regions was performed using multiple images (n=12 for WT cells; n=10 for MON2-KO cells) (Fig. 4b). The Pearson’s correlation coefficient (PCC) for EGFP-RAB4B versus Scarlet-Giantin changed significantly from 0.691±0.029 in WT cells to 0.405±0.037 in MON2-KO cells, suggesting that RAB4B-positive RE near the Golgi region decreased in MON2-KO cells. The re-distribution of RAB4B to the Golgi was rescued by expressing TagRFP-MON2 in MON2-KO cells (Supplemental Figure S4b), demonstrating the functionality of TagRFP-MON2 and that RAB4B mislocalization is caused by MON2 deletion.

To determine the contributions of DOPEY proteins to RE distribution, we next analyzed the localization of RAB4B in DOPEY1-KO, DOPEY2-KO, and DOPEY1 and DOPEY2 double-KO (D-KO) cells. RAB4B localization was only minimally affected in DOPEY1-KO cells. Unexpectedly, in DOPEY2-KO and D-KO cells, RAB4B localization increased around the Golgi and disappeared in the peripheral dots and around EE membrane (Supplemental Figure S4c). To verify the RAB4B localization more clearly, cells were treated with nocodazole to introduce mini-stacks. After nocodazole treatment, RAB4B was co-localized with Giantin in D-KO cells, but was concentrated in SNX3-positive structures in MON2-KO cells (Supplemental Figure S4d). These results demonstrate that deletion of MON2 and DOPEY have distinct phenotypes on RE localization and therefore suggest a more complex relationship than expected (See Discussion part).

In mammalian cells, RE are also present as dense tubular networks (Xie *et al.*, 2016). To determine if MON2 contributes to the formation of this tubular network, we compared the localization of RAB4B-positive RE in WT and in MON2-KO cells by live-cell imaging. Reticular-like networks were readily observed and the tubules showed dynamic movement in WT cells (Movie 3). In contrast the dynamic tubular networks were lost in MON2-KO cells. These results indicate that MON2 is required for tubularization of RE at the site of EE and regulates the movement of RE.

### MON2 is required for recycling of transferrin receptor

**Fig. 5:**
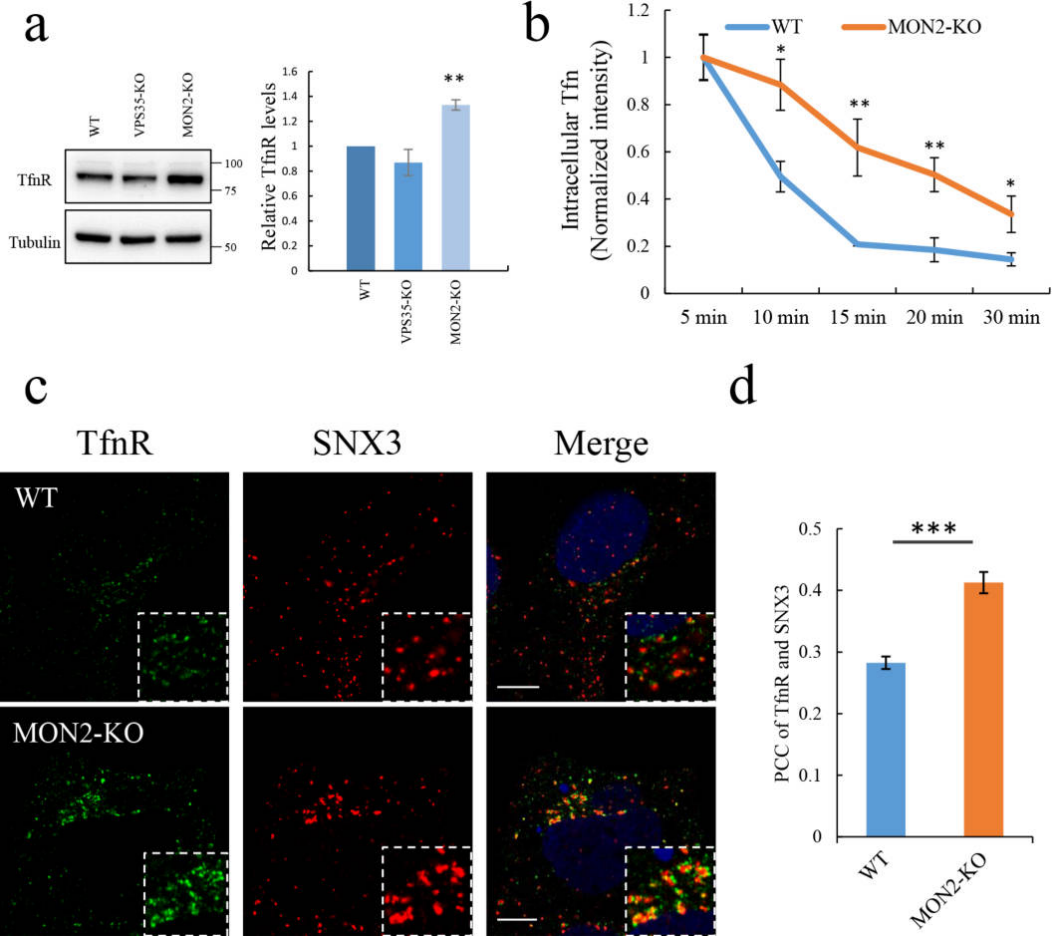
MON2 is required for optimal TfnR recycling. a, Immunoblotting of endogenous TfnR in HEK293 (WT), VPS35-KO and MON2-KO cells (left). Tubulin was used as a loading control. Quantification of TfnR in indicated cells (right). Data represent means±SE (n=3, **, *P*<0.01). b, HEK293 (WT) or MON2-KO cells were incubated with CF488A-Tfn at 4°C for 1 h, and then chased at 37°C. Data represent means±SE, with normalization by the cell number in each field. *, *P*<0.05; **, *P*<0.01, compared with control cells. c, Endogenous TfnR and SNX3 were immunostained in HEK293 (WT) and MON2-KO cells. Scale bars, 10 μm. d, Quantification of co-localization of TfnR with SNX3 by PCC. Data represent means±SE, ***, *P*<0.001 (two-tailed Student’s *t* test).

The requirement of MON2 for maintenance of RE structures prompted us to investigate if MON2 plays a role in endocytic recycling. One of well-known RE marker is transferrin receptor (TfnR), which continuously recycles between PM and endocytic system (Kobayashi and Fukuda, 2013; Maxfield and McGraw, 2004). When endocytic recycling is impaired, the steady state level of TfnR is altered. We analyzed the TfnR level in MON2-KO and VPS35-KO cells. The steady-state levels of TfnR do not depend on VPS35 (Kvainickas *et al.*, 2017). As shown in Fig. 5a, the levels of TfnR was unaffected in VPS35-KO cells. In contrast, the steady-state levels of TfnR were increased in MON2-KO cells, suggesting that the MON2 is affected the TfnR recycling. To further investigate this phenotype, fluorescently-labeled transferrin (Tfn-488) was internalized for 1 h at 4°C in WT and MON2-KO cells, after which the cells were chased and quantified for their intracellular fluorescence. The intracellular fluorescence of Tfn-488 decreased more slowly in MON2-KO cells (t_1/2_=~20 min) than in WT cells (t_1/2_=~10 min) (Fig. 5b) suggesting the increased levels of TfnR is due to its increased intracellular retention. We next checked the localization of endogenous TfnR by compared with EE marker SNX3 in WT and MON2-KO cells. In WT cells, TfnR-positive dots were separated from SNX3-positive EE (Fig. 5c). In contrast, TfnR was accumulated around EE in MON2-KO cells. Quantitative analysis revealed that the co-localization of TfnR with SNX3 was increased (Fig. 5d), suggesting that MON2 is required for efficient recycling of TfnR.

### Wls passes through RE for retrograde transport to the Golgi

**Fig. 6:**
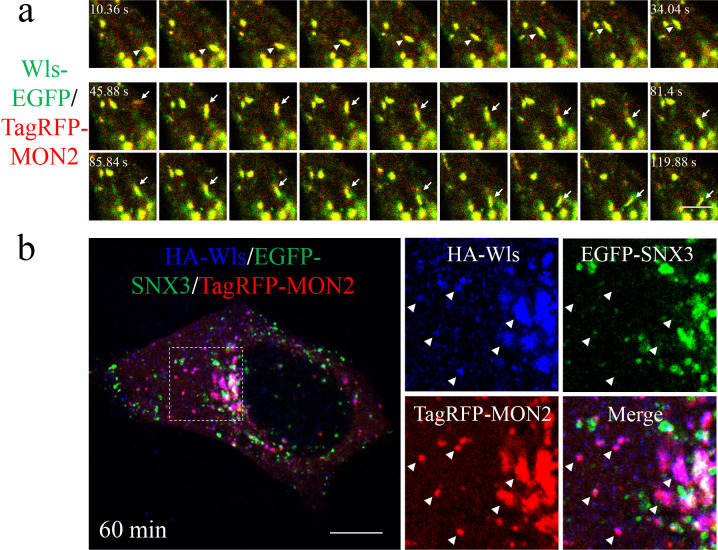
Endocytic Wls passes through RE. a, Time-lapse images from a video (Movie 4) of a cell co-expressing TagRFP-MON2 and Wls-EGFP. The arrowheads and arrows indicate Wls migrating with MON2. Scale bar, 5 μm. b, Representative confocal images of HA-Wls, EGFP-SNX3, and TagRFP-MON2 in HEK293 cells. The enlarged images show magnified views of the boxed areas. After a 60-min chase, the localization of surface-labeled HA-Wls was observed. The arrowheads indicate HA-Wls that localized at MON2-positive endosomes, in which SNX3-labeled EEs were absent. Scale bar, 10 μm.

Wls is a critical cargo receptor for the secretion of Wnt proteins (Banziger *et al.*, 2006), and undergoes continuous recycling between the PM, EE, and Golgi (Gasnereau *et al.*, 2011; Port *et al.*, 2008). In EE, the SNX3-retromer complex is needed for recognition and enrichment of Wls (Harterink *et al.*, 2011; Zhang *et al.*, 2011). MON2 is required for Wls recycling (McGough *et al.*, 2018) but at which specific steps remains to be determined. We hypothesized that the MON2-DOPEY complex mediates abscission of Wls-containing vesicular carriers from EE. To test this hypothesis, we analyzed the steady-state localizations of Wls-EGFP in the Golgi (marked with Scarlet-Giantin), EE (marked with TagBFP-SNX3), and RE (marked with TagRFP-MON2). Wls-EGFP was primarily localized to the Golgi in WT cells. Both SNX3- and MON2-positive endosomes contained Wls-EGFP signals (Supplemental Figure S5). Furthermore, the compartments containing both Wls-EGFP and TagRFP-MON2 exhibited co-movement during live-cell imaging (Fig. 6a, Movie 4), suggesting that MON2-positive RE contain Wls as cargo molecules.

Is endocytosed Wls transported from EE to the Golgi directly, or does it pass through RE? To determine the route of the PM-localized Wls, we took advantage of an HA-tagged Wls construct (HA-Wls), in which an HA-tag was inserted in the first extracellular loop (Varandas *et al.*, 2016). WT cells expressing HA-Wls were labeled with an anti-HA antibody for 15 min, after which the cells were chased in normal medium. HA-Wls accumulated at SNX3-labeled EE after a 15-min chase (data not shown). After a 60-min chase, HA-Wls was mainly co-localized with MON2-positive RE (Fig. 6b), suggesting that HA-Wls was transported to RE after its presence in EE.

**Fig. 7:**
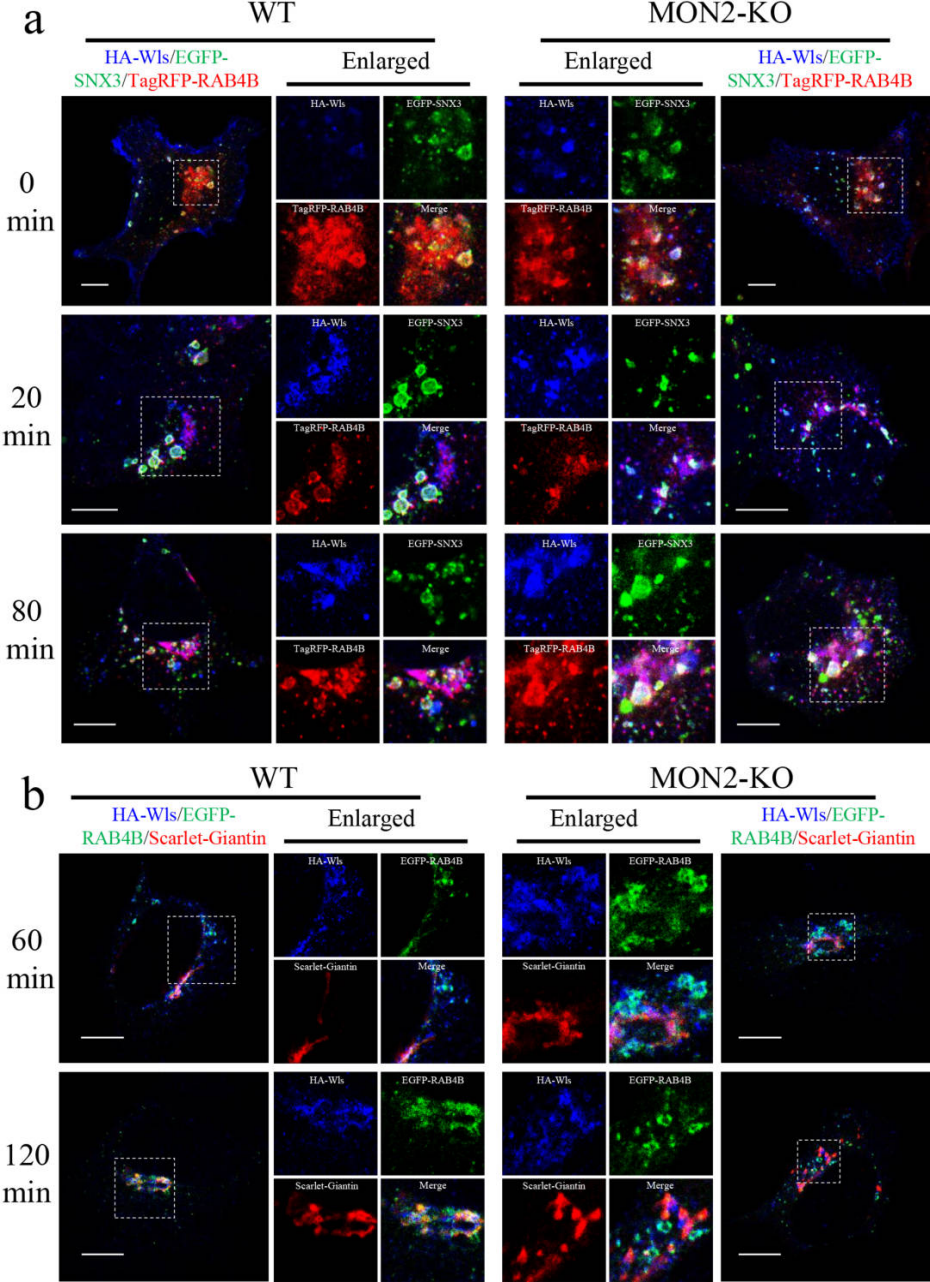
Retrograde transport of Wls to the Golgi is impaired in MON2-KO cells. a, Representative confocal images of HA-Wls, EGFP-SNX3, and TagRFP-RAB4B in HEK293 WT or MON2-KO cells. Enlarged images show magnified views of the boxed areas. Images were taken at the indicated times after chasing of surface-labeled HA-Wls. Scale bars, 10 μm. See Supplemental Figure S6a and b for quantitative analyses. b, Representative confocal images of HA-Wls, EGFP-RAB4B, and Scarlet-Giantin in HEK293 WT or MON2-KO cells. The enlarged images show magnified views of the boxed areas. Images were taken at the indicated times after chasing of surfaced-labeled HA-Wls. Scale bars, 10 μm. See Supplemental Figure S6c and d for quantitative analyses.

The observation that endocytosed HA-Wls passes through RE prompted us to examine the requirement of MON2 for transport of Wls from EE to the Golgi. After Wls internalization, its localization was examined by microscopy in WT and MON2-KO cells. EGFP-SNX3 and TagRFP-RAB4B were used as an EE and RE marker, respectively. Initially, HA-Wls was mainly localized to the PM and partly at SNX3-positive EE in both WT and MON2-KO cells (Fig. 7a). After a 20-min chase, HA-Wls started to appear at both EE and RE, with an almost equal distribution. After an 80-min chase, the localization of HA-Wls at SNX3-positive EE was reduced in WT cells, whereas HA-Wls in RAB4B-positive RE remained constant. In MON2-KO cells, however, HA-Wls remained localized at SNX3-positive EE (Fig. 7a). Furthermore, the localization of HA-Wls in RAB4B-positive RE continuously increased (Supplemental Figure S6a, b). These results demonstrate that Wls is accumulated at EE and RE in MON2-KO cells.

Next, transport of HA-Wls to the Golgi was examined. We labeled RE and the Golgi with EGFP-RAB4B and Scarlet-Giantin, respectively. Notably, RAB4B localization that was predominantly around the Golgi in WT cells was decreased in MON2-KO cells (Fig. 4a). After chasing for 60 and 120 min, HA-Wls was predominantly localized to the Golgi in WT cells but remained co-localized with RAB4B-positive compartments and barely appeared in the Giantin-positive Golgi in MON2-KO cells (Fig. 7b). Quantitative analysis further revealed that the localization of HA-Wls with Giantin was not increased in MON2-KO cells (Supplemental Figure S6c, d). Taken together, these results indicate that transport of Wls from RE to the Golgi is impaired by MON2 deletion.

### The steady state of Wls-EGFP is mis-localized to lysosome in MON2-KO cells

To analyze where Wls is trafficked in MON2-KO cells, we examined its steady state levels and localization. It is reported that deletion of VPS35 causes Wls mis-localization to lysosomes (Port *et al.*, 2008; Yang *et al.*, 2008). Wls-EGFP localization was analyzed with the endogenous Golgi marker Giantin and lysosome marker LAMP1. In WT cells, Wls-EGFP was mainly localized at the Golgi (Supplemental Figure S7a). In contrast, intracellular punctate fluorescent signals of Wls-EGFP appeared in VPS35-KO and MON2-KO cells. These punctate structures co-localized with the lysosomal marker LAMP1. Quantitative analysis of Wls-EGFP revealed that the co-localization of Wls-GFP with Giantin was significantly reduced in MON2-KO and VPS35-KO cells (Supplemental Figure S7b), while that with LAMP1 was increased (Supplemental Figure S7c). To confirm the result, we analyzed the localization of HA-Wls at 120 min after starting the endocytosis. The localization of HA-Wls was compared with that of LAMP1. In WT cells, HA-Wls was accumulated at Golgi-like structures (Fig. 7b), whereas it did not colocalize with LAMP1 (Supplemental Figure S7d). In MON2-KO cells, HA-Wls started to co-localize with LAMP1 partially (Supplemental Figure S7d). Taken together, these results indicate that Wls is missorted to lysosomes when MON2 is deleted.

## Discussion

**Fig. 8:**
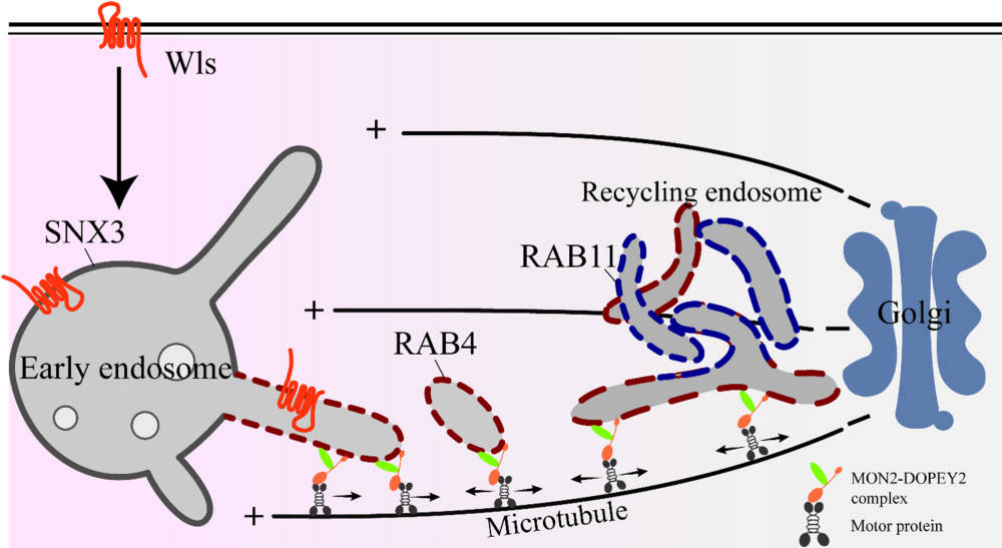
Summary model that MON2-DOPEY complex guides Wls transport to the Golgi through RE. At EE, cargoes such as Wls were incorporated to the carrier formed through SNX3 and ATP9A function (McGough *et al.*, 2018). Pre-existing RE approach and contact with the EE to take newly synthesized RE tubules through the DOPEY protein function, then separate from EE through bidirectional movement mediated by MON2-DOPEY complex.

In the present study, we demonstrate that the MON2 regulates RE distribution and movement, which are required for retrograde transport of Wls from EE to the Golgi (Fig. 8). In MON2-KO cells, RAB4B-positive RE were retained around EE. As a consequence, recycling of endocytic cargos like Wls and TfnR, which pass through RE, was impaired.

MON2 was first reported to localize at the TGN in HeLa cells (Mahajan *et al.*, 2013), although a subsequent study revealed that endogenous MON2 may reside in both the Golgi and RE (Mahajan *et al.*, 2019). In the present study, we carefully checked MON2 localization and found that both EGFP and TagRFP-tagged MON2 localized at perinuclear regions as well as in peripheral dots. The Golgi and perinuclear RE tubules are both concentrated at the MTOC in cells (Saraste and Prydz, 2019). Analysis of mini-stacks introduced by nocodazole revealed that MON2, like RE markers, was localized at trans-side of the stacks (Fujii *et al.*, 2020). Both MON2-positive perinuclear regions and peripheral dots were merged with RE markers, suggesting that MON2 localizes at RE.

We found that both DOPEY1 and DOPEY2 interacted with MON2. Furthermore, ectopic MON2 expression shifted the cytoplasmic localization of DOPEY1 and DOPEY2 to RE localization, demonstrating that MON2 recruits both DOPEY1 and DOPEY2 to the RE membrane. Although MON2 forms a complex with both DOPEY1 and DOPEY2, the localization of RAB4B differed in each of the deletion cells (Fig. 4a and Supplemental Figure S4a). One possible explanation for these markedly different phenotypes is that the bidirectional transport of RE. The N-terminal of DOPEY1 interacts with a motor protein, kinesin-1 (Mahajan *et al.*, 2019). Our preliminary experiment also showed that MON2-DOPEY has binding abilities to kinesin and dynein/dynactin motor proteins (Supplemental Figure S8), suggesting that the MON2-DOPEY complex drives the RE movement through motor proteins. In DOPEY-KO cells, pre-existing RE would fail to move toward EE so accumulate at Golgi. On the other hand, MON2 might regulate DOPEY-motor activity at the site of RE generation on EE. In MON2-KO cells, failure to segregate newly synthesized tubular RE from EE results in Wls accumulation near EE structures (Fig. 8).

Recycling of Wls was impaired in MON2-KO cells. Wls continually undergoes recycling between the plasma membrane, EE and the Golgi (Harterink *et al.*, 2011; Port *et al.*, 2008), whereas it has remained unclear whether Wls is directly transported from EE to the Golgi or through RE. We showed that endocytic Wls is transported to MON2- and RAB4B-positive RE, indicating that Wls is recycled back to the Golgi through RE. The SNX3-retromer complex is essential for the recognition of Wls and ATP9A is responsible for the membrane deformation that is required for carrier formation (McGough *et al.*, 2018). It has been reported that ATP9A is also localized at RE (Tanaka *et al.*, 2016). The MON2-DOPEY complex associates with ATP9A and SNX3 (McGough *et al.*, 2018), and could play roles in carrier transport.

On the other hand, knockdown of MON2 accelerates the transport of Furin and CI-M6PR from endosome to TGN (Mahajan *et al.*, 2013). Furin is trafficked to the TGN directly through late endosomes without passing RE compartment (Mallet and Maxfield, 1999), and CI-M6PR is a cargo of SNX-BAR carriers (Kvainickas *et al.*, 2017). Compared with the long tubular carriers formed by SNX-BAR, the membrane carriers containing Wls, which are formed by the ATP9A-SNX3-retromer, are vesicular (Harterink *et al.*, 2011). The evidence support that retrograde transport of Furin and CI-M6PR is independent of MON2 function. Depletion of MON2 causes SNX3-retromer blockade and may accelerate the transport of Furin and CI-M6PR to the Golgi due to upregulation of other retrograde pathways such as SNX-BAR.

It is well established that several mechanisms including actin-driven, dynein-driven, and ER-endosome contact site-driven processes are involved in the fission of SNX-BAR tubules from LE (Daly and Cullen, 2018). However, less is known about how the SNX3-retromer complex carriers are excised from EE. Under live-cell imaging, we observed that peripheral MON2-positive RE approached SNX3-labeled EE, displayed a transient interaction, and then separated from them. This behavior is similar to the ‘hug-and-kiss’ model proposed for receipt of COPII vesicles by the *cis*-Golgi at the endoplasmic reticulum exit sites (Kurokawa *et al.*, 2014). One model that our data support is that pre-existing RE interacts with newly synthesized RE at the tubular EE through the bidirectional movement on microtubules, and Wls may be incorporated from EE to RE. Once Wls has been transported to RE, it is delivered to the TGN. These possibilities will be addressed in future investigations.

## Conflict of interest

The authors declare that they have no conflicts of interest with the contents of this article.

## Abbreviations

D-KO: DOPEY1 and DOPEY2 double-knockout
EE: early endosomes
HEK293: human embryonic kidney 293
KO: knockout
LE: late endosomes
MCC: Manders correlation coefficients
PCC: Pearson’s correlation coefficient
PM: plasma membrane
RE: recycling endosomes
SNX3: sorting nexin 3
TGN: *trans*-Golgi network
Wls: Wntless
WT: wild-type

## References

[B1] Bai, Z. and Grant, B.D. 2015. A TOCA/CDC-42/PAR/WAVE functional module required for retrograde endocytic recycling. Proc. Natl. Acad. Sci. USA, 112: E1443–1452. 25775511 10.1073/pnas.1418651112 PMC4378436

[B2] Banziger, C., Soldini, D., Schutt, C., Zipperlen, P., Hausmann, G., and Basler, K. 2006. Wntless, a conserved membrane protein dedicated to the secretion of Wnt proteins from signaling cells. Cell, 125: 509–522. 16678095 10.1016/j.cell.2006.02.049

[B3] Barbosa, S., Pratte, D., Schwarz, H., Pipkorn, R., and Singer-Kruger, B. 2010. Oligomeric Dop1p is part of the endosomal Neo1p-Ysl2p-Arl1p membrane remodeling complex. Traffic, 11: 1092–1106. 20477991 10.1111/j.1600-0854.2010.01079.x

[B4] Bhattacharyya, D., Hammond, A.T., and Glick, B.S. 2010. High-quality immunofluorescence of cultured cells. Methods Mol. Biol., 619: 403–410. 20419424 10.1007/978-1-60327-412-8_24 PMC2893412

[B5] Bolte, S. and Cordelieres, F.P. 2006. A guided tour into subcellular colocalization analysis in light microscopy. J. Microsc., 224: 213–232. 17210054 10.1111/j.1365-2818.2006.01706.x

[B6] Cullen, P.J. and Steinberg, F. 2018. To degrade or not to degrade: mechanisms and significance of endocytic recycling. Nat. Rev. Mol. Cell Biol., 19: 679–696. 30194414 10.1038/s41580-018-0053-7

[B7] D’Souza, R.S., Semus, R., Billings, E.A., Meyer, C.B., Conger, K., and Casanova, J.E. 2014. Rab4 orchestrates a small GTPase cascade for recruitment of adaptor proteins to early endosomes. Curr. Biol., 24: 1187–1198. 24835460 10.1016/j.cub.2014.04.003 PMC4059052

[B8] Daly, J.L. and Cullen, P.J. 2018. Endoplasmic Reticulum-Endosome Contact Sites: Specialized Interfaces for Orchestrating Endosomal Tubule Fission? Biochemistry, 57: 6738–6740. 30500172 10.1021/acs.biochem.8b01176

[B9] Day, K.J., Casler, J.C., and Glick, B.S. 2018. Budding Yeast Has a Minimal Endomembrane System. Dev. Cell, 44: 56–72 e54. 29316441 10.1016/j.devcel.2017.12.014 PMC5765772

[B10] de Wit, H., Lichtenstein, Y., Kelly, R.B., Geuze, H.J., Klumperman, J., and van der Sluijs, P. 2001. Rab4 regulates formation of synaptic-like microvesicles from early endosomes in PC12 cells. Mol. Biol. Cell, 12: 3703–3715. 11694600 10.1091/mbc.12.11.3703 PMC60287

[B11] Delevoye, C., Miserey-Lenkei, S., Montagnac, G., Gilles-Marsens, F., Paul-Gilloteaux, P., Giordano, F., Waharte, F., Marks, M.S., Goud, B., and Raposo, G. 2014. Recycling endosome tubule morphogenesis from sorting endosomes requires the kinesin motor KIF13A. Cell Rep., 6: 445–454. 24462287 10.1016/j.celrep.2014.01.002 PMC3928541

[B12] Efe, J.A., Plattner, F., Hulo, N., Kressler, D., Emr, S.D., and Deloche, O. 2005. Yeast Mon2p is a highly conserved protein that functions in the cytoplasm-to-vacuole transport pathway and is required for Golgi homeostasis. J. Cell Sci., 118: 4751–4764. 16219684 10.1242/jcs.02599

[B13] Etoh, K. and Fukuda, M. 2019. Rab10 regulates tubular endosome formation through KIF13A and KIF13B motors. J. Cell Sci., 132: jcs226977. 10.1242/jcs.226977 30700496

[B14] Fujii, S., Kurokawa, K., Inaba, R., Hiramatsu, N., Tago, T., Nakamura, Y., Nakano, A., Satoh, T., and Satoh, A.K. 2020. Recycling endosomes attach to the trans-side of Golgi stacks in Drosophila and mammalian cells. J. Cell Sci., 133: jcs236935. 10.1242/jcs.236935 31974113

[B15] Gasnereau, I., Herr, P., Chia, P.Z., Basler, K., and Gleeson, P.A. 2011. Identification of an endocytosis motif in an intracellular loop of Wntless protein, essential for its recycling and the control of Wnt protein signaling. J. Biol. Chem., 286: 43324–43333. 22027831 10.1074/jbc.M111.307231 PMC3234804

[B16] Gautreau, A., Oguievetskaia, K., and Ungermann, C. 2014. Function and regulation of the endosomal fusion and fission machineries. Cold Spring Harb. Perspect. Biol., 6: a016832. 10.1101/cshperspect.a016832 PMC3949357 24591520

[B17] Gillingham, A.K., Whyte, J.R., Panic, B., and Munro, S. 2006. Mon2, a relative of large Arf exchange factors, recruits Dop1 to the Golgi apparatus. J. Biol. Chem., 281: 2273–2280. 16301316 10.1074/jbc.M510176200

[B18] Grant, B.D. and Donaldson, J.G. 2009. Pathways and mechanisms of endocytic recycling. Nat. Rev. Mol. Cell Biol., 10: 597–608. 19696797 10.1038/nrm2755 PMC3038567

[B19] Green, E.G., Ramm, E., Riley, N.M., Spiro, D.J., Goldenring, J.R., and Wessling-Resnick, M. 1997. Rab11 is associated with transferrin-containing recycling compartments in K562 cells. Biochem. Biophys. Res. Commun., 239: 612–616. 9344879 10.1006/bbrc.1997.7520

[B20] Harterink, M., Port, F., Lorenowicz, M.J., McGough, I.J., Silhankova, M., Betist, M.C., van Weering, J.R.T., van Heesbeen, R., Middelkoop, T.C., Basler, K., Cullen, P.J., and Korswagen, H.C. 2011. A SNX3-dependent retromer pathway mediates retrograde transport of the Wnt sorting receptor Wntless and is required for Wnt secretion. Nat. Cell Biol., 13: 914–923. 21725319 10.1038/ncb2281 PMC4052212

[B21] Hirokawa, N., Noda, Y., Tanaka, Y., and Niwa, S. 2009. Kinesin superfamily motor proteins and intracellular transport. Nat. Rev. Mol. Cell Biol., 10: 682–696. 19773780 10.1038/nrm2774

[B22] Hirota, Y., Kuronita, T., Fujita, H., and Tanaka, Y. 2007. A role for Rab5 activity in the biogenesis of endosomal and lysosomal compartments. Biochem. Biophys. Res. Commun., 364: 40–47. 17927960 10.1016/j.bbrc.2007.09.089

[B23] Jovic, M., Sharma, M., Rahajeng, J., and Caplan, S. 2010. The early endosome: a busy sorting station for proteins at the crossroads. Histol. Histopathol., 25: 99–112. 19924646 10.14670/hh-25.99 PMC2810677

[B24] Klumperman, J. and Raposo, G. 2014. The complex ultrastructure of the endolysosomal system. Cold Spring Harb. Perspect. Biol., 6: a016857. 24851870 10.1101/cshperspect.a016857 PMC4176003

[B25] Kobayashi, H. and Fukuda, M. 2013. Arf6, Rab11 and transferrin receptor define distinct populations of recycling endosomes. Commun. Integr. Biol., 6: e25036. 24255739 10.4161/cib.25036 PMC3829897

[B26] Kurokawa, K., Okamoto, M., and Nakano, A. 2014. Contact of cis-Golgi with ER exit sites executes cargo capture and delivery from the ER. Nat. Commun., 5: 3653. 24728174 10.1038/ncomms4653 PMC3996532

[B27] Kvainickas, A., Jimenez-Orgaz, A., Nagele, H., Hu, Z., Dengjel, J., and Steinberg, F. 2017. Cargo-selective SNX-BAR proteins mediate retromer trimer independent retrograde transport. J. Cell Biol., 216: 3677–3693. 28935632 10.1083/jcb.201702137 PMC5674888

[B28] Liu, Y.S., Guo, X.Y., Hirata, T., Rong, Y., Motooka, D., Kitajima, T., Murakami, Y., Gao, X.D., Nakamura, S., Kinoshita, T., and Fujita, M. 2018. N-Glycan-dependent protein folding and endoplasmic reticulum retention regulate GPI-anchor processing. J. Cell Biol., 217: 585–599. 29255114 10.1083/jcb.201706135 PMC5800811

[B29] Mahajan, D., Boh, B.K., Zhou, Y., Chen, L., Cornvik, T.C., Hong, W., and Lu, L. 2013. Mammalian Mon2/Ysl2 regulates endosome-to-Golgi trafficking but possesses no guanine nucleotide exchange activity toward Arl1 GTPase. Sci Rep, 3: 3362. 24285343 10.1038/srep03362 PMC3842536

[B30] Mahajan, D., Tie, H.C., Chen, B., and Lu, L. 2019. Dopey1-Mon2 complex binds to dual-lipids and recruits kinesin-1 for membrane trafficking. Nat. Commun., 10: 3218. 31324769 10.1038/s41467-019-11056-5 PMC6642134

[B31] Mallet, W.G. and Maxfield, F.R. 1999. Chimeric forms of furin and TGN38 are transported with the plasma membrane in the trans-Golgi network via distinct endosomal pathways. J. Cell Biol., 146: 345–359. 10465644 10.1083/jcb.146.2.345 PMC2156176

[B32] Martin, M. and Akhmanova, A. 2018. Coming into Focus: Mechanisms of Microtubule Minus-End Organization. Trends Cell Biol., 28: 574–588. 29571882 10.1016/j.tcb.2018.02.011

[B33] Maxfield, F.R. and McGraw, T.E. 2004. Endocytic recycling. Nat. Rev. Mol. Cell Biol., 5: 121–132. 15040445 10.1038/nrm1315

[B34] McGough, I.J., de Groot, R.E.A., Jellett, A.P., Betist, M.C., Varandas, K.C., Danson, C.M., Heesom, K.J., Korswagen, H.C., and Cullen, P.J. 2018. SNX3-retromer requires an evolutionary conserved MON2:DOPEY2:ATP9A complex to mediate Wntless sorting and Wnt secretion. Nat. Commun., 9: 3737. 30213940 10.1038/s41467-018-06114-3 PMC6137200

[B35] Perrin, L., Lacas-Gervais, S., Gilleron, J., Ceppo, F., Prodon, F., Benmerah, A., Tanti, J.F., and Cormont, M. 2013. Rab4b controls an early endosome sorting event by interacting with the gamma-subunit of the clathrin adaptor complex 1. J. Cell Sci., 126: 4950–4962. 24006255 10.1242/jcs.130575

[B36] Pfeffer, S.R. 2013. Rab GTPase regulation of membrane identity. Curr. Opin. Cell Biol., 25: 414–419. 23639309 10.1016/j.ceb.2013.04.002 PMC3729790

[B37] Pfeffer, S.R. 2017. Rab GTPases: master regulators that establish the secretory and endocytic pathways. Mol. Biol. Cell, 28: 712–715. 28292916 10.1091/mbc.E16-10-0737 PMC5349778

[B38] Port, F., Kuster, M., Herr, P., Furger, E., Banziger, C., Hausmann, G., and Basler, K. 2008. Wingless secretion promotes and requires retromer-dependent cycling of Wntless. Nat. Cell Biol., 10: 178–185. 18193032 10.1038/ncb1687

[B39] Purushothaman, L.K. and Ungermann, C. 2018. Cargo induces retromer-mediated membrane remodeling on membranes. Mol. Biol. Cell, 29: 2709–2719. 30188774 10.1091/mbc.E18-06-0339 PMC6249844

[B40] Ren, M., Xu, G., Zeng, J., De Lemos-Chiarandini, C., Adesnik, M., and Sabatini, D.D. 1998. Hydrolysis of GTP on rab11 is required for the direct delivery of transferrin from the pericentriolar recycling compartment to the cell surface but not from sorting endosomes. Proc. Natl. Acad. Sci. USA, 95: 6187–6192. 9600939 10.1073/pnas.95.11.6187 PMC27621

[B41] Saraste, J. and Prydz, K. 2019. A New Look at the Functional Organization of the Golgi Ribbon. Front. Cell Dev. Biol., 7: 171. 31497600 10.3389/fcell.2019.00171 PMC6713163

[B42] Scott, C.C., Vacca, F., and Gruenberg, J. 2014. Endosome maturation, transport and functions. Semin. Cell Dev. Biol., 31: 2–10. 24709024 10.1016/j.semcdb.2014.03.034

[B43] Shakya, S., Sharma, P., Bhatt, A.M., Jani, R.A., Delevoye, C., and Setty, S.R. 2018. Rab22A recruits BLOC-1 and BLOC-2 to promote the biogenesis of recycling endosomes. EMBO Rep., 19: e45918. 10.15252/embr.201845918 PMC6280653 30404817

[B44] Sonnichsen, B., De Renzis, S., Nielsen, E., Rietdorf, J., and Zerial, M. 2000. Distinct membrane domains on endosomes in the recycling pathway visualized by multicolor imaging of Rab4, Rab5, and Rab11. J. Cell Biol., 149: 901–914. 10811830 10.1083/jcb.149.4.901 PMC2174575

[B45] Taguchi, T. 2013. Emerging roles of recycling endosomes. J. Biochem., 153: 505–510. 23625997 10.1093/jb/mvt034

[B46] Tanaka, Y., Ono, N., Shima, T., Tanaka, G., Katoh, Y., Nakayama, K., Takatsu, H., and Shin, H.W. 2016. The phospholipid flippase ATP9A is required for the recycling pathway from the endosomes to the plasma membrane. Mol. Biol. Cell, 27: 3883–3893. 27733620 10.1091/mbc.E16-08-0586 PMC5170610

[B47] Ullrich, O., Reinsch, S., Urbe, S., Zerial, M., and Parton, R.G. 1996. Rab11 regulates recycling through the pericentriolar recycling endosome. J. Cell Biol., 135: 913–924. 8922376 10.1083/jcb.135.4.913 PMC2133374

[B48] Varandas, K.C., Irannejad, R., and von Zastrow, M. 2016. Retromer Endosome Exit Domains Serve Multiple Trafficking Destinations and Regulate Local G Protein Activation by GPCRs. Curr. Biol., 26: 3129–3142. 27839977 10.1016/j.cub.2016.09.052 PMC5140749

[B49] Wilson, J.M., de Hoop, M., Zorzi, N., Toh, B.H., Dotti, C.G., and Parton, R.G. 2000. EEA1, a tethering protein of the early sorting endosome, shows a polarized distribution in hippocampal neurons, epithelial cells, and fibroblasts. Mol. Biol. Cell, 11: 2657–2671. 10930461 10.1091/mbc.11.8.2657 PMC14947

[B50] Xie, S., Bahl, K., Reinecke, J.B., Hammond, G.R., Naslavsky, N., and Caplan, S. 2016. The endocytic recycling compartment maintains cargo segregation acquired upon exit from the sorting endosome. Mol. Biol. Cell, 27: 108–126. 26510502 10.1091/mbc.E15-07-0514 PMC4694750

[B51] Xu, Y., Hortsman, H., Seet, L., Wong, S.H., and Hong, W. 2001. SNX3 regulates endosomal function through its PX-domain-mediated interaction with PtdIns(3)P. Nat. Cell Biol., 3: 658–666. 11433298 10.1038/35083051

[B52] Yang, P.T., Lorenowicz, M.J., Silhankova, M., Coudreuse, D.Y., Betist, M.C., and Korswagen, H.C. 2008. Wnt signaling requires retromer-dependent recycling of MIG-14/Wntless in Wnt-producing cells. Dev. Cell, 14: 140–147. 18160347 10.1016/j.devcel.2007.12.004

[B53] Zhang, P., Wu, Y., Belenkaya, T.Y., and Lin, X. 2011. SNX3 controls Wingless/Wnt secretion through regulating retromer-dependent recycling of Wntless. Cell Res., 21: 1677–1690. 22041890 10.1038/cr.2011.167 PMC3357989

[B54] Zhao, S.B., Suda, Y., Nakanishi, H., Wang, N., Yoko, O.T., Gao, X.D., and Fujita, M. 2019. Yeast Dop1 is required for glycosyltransferase retrieval from the trans-Golgi network. Biochim. Biophys. Acta. Gen. Subj., 1863: 1147–1157. 30981741 10.1016/j.bbagen.2019.04.009

