# Supplementary figures and images for: MON2 Guides Wntless Transport to the Golgi through Recycling Endosomes
Source: Cell Struct Funct. 2020 May 12;45(1):77–92. doi: 10.1247/csf.20012 (PMC10511057; doi:10.1247/csf.20012)

# Supplemental Figure S1

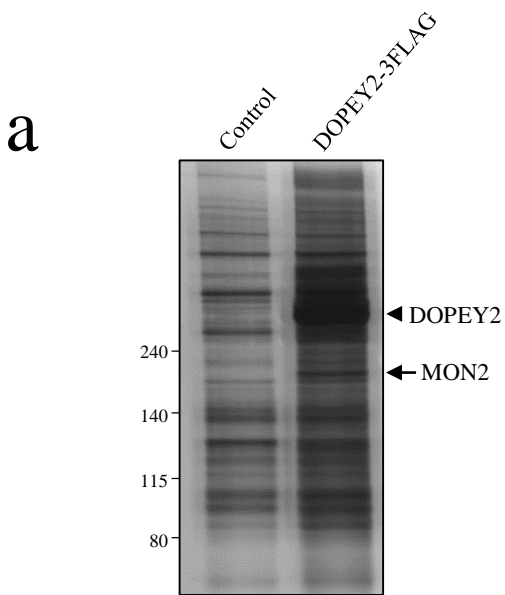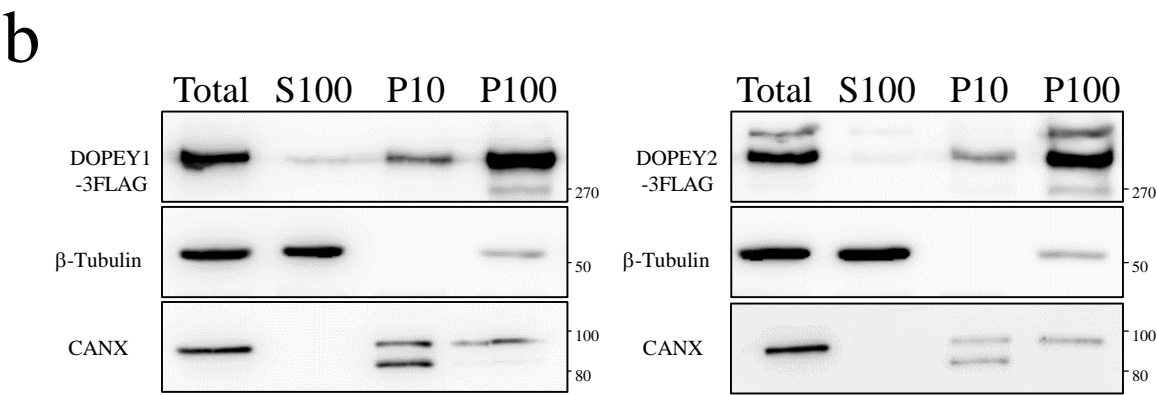

Supplement: Supplementary file 1 — Supplemental Figure S1 [file csf_45_20012_1.pdf]

# Supplemental Figure S3

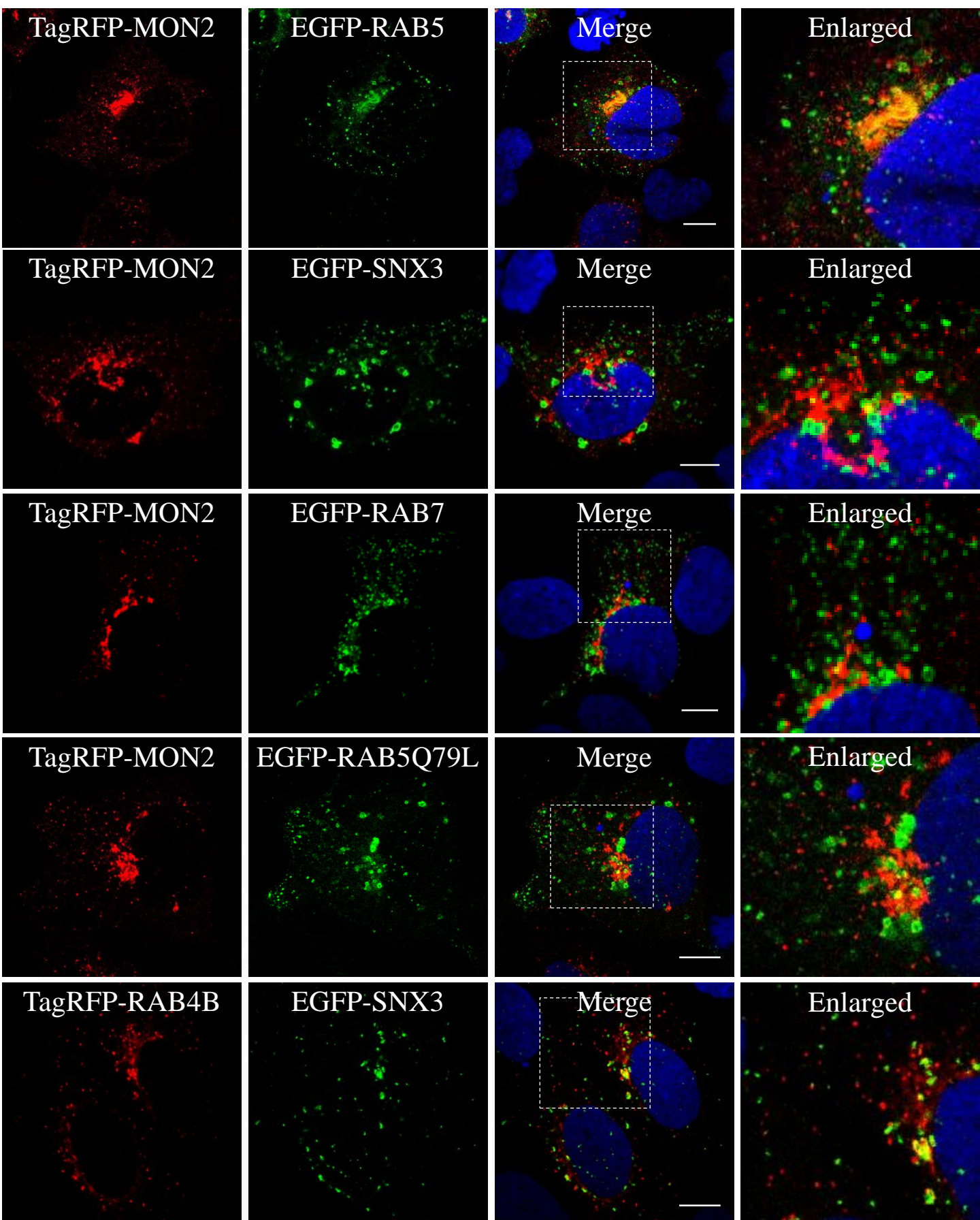

Supplement: Supplementary file 3 — Supplemental Figure S3 [file csf_45_20012_3.pdf]

# Supplemental Figure S5

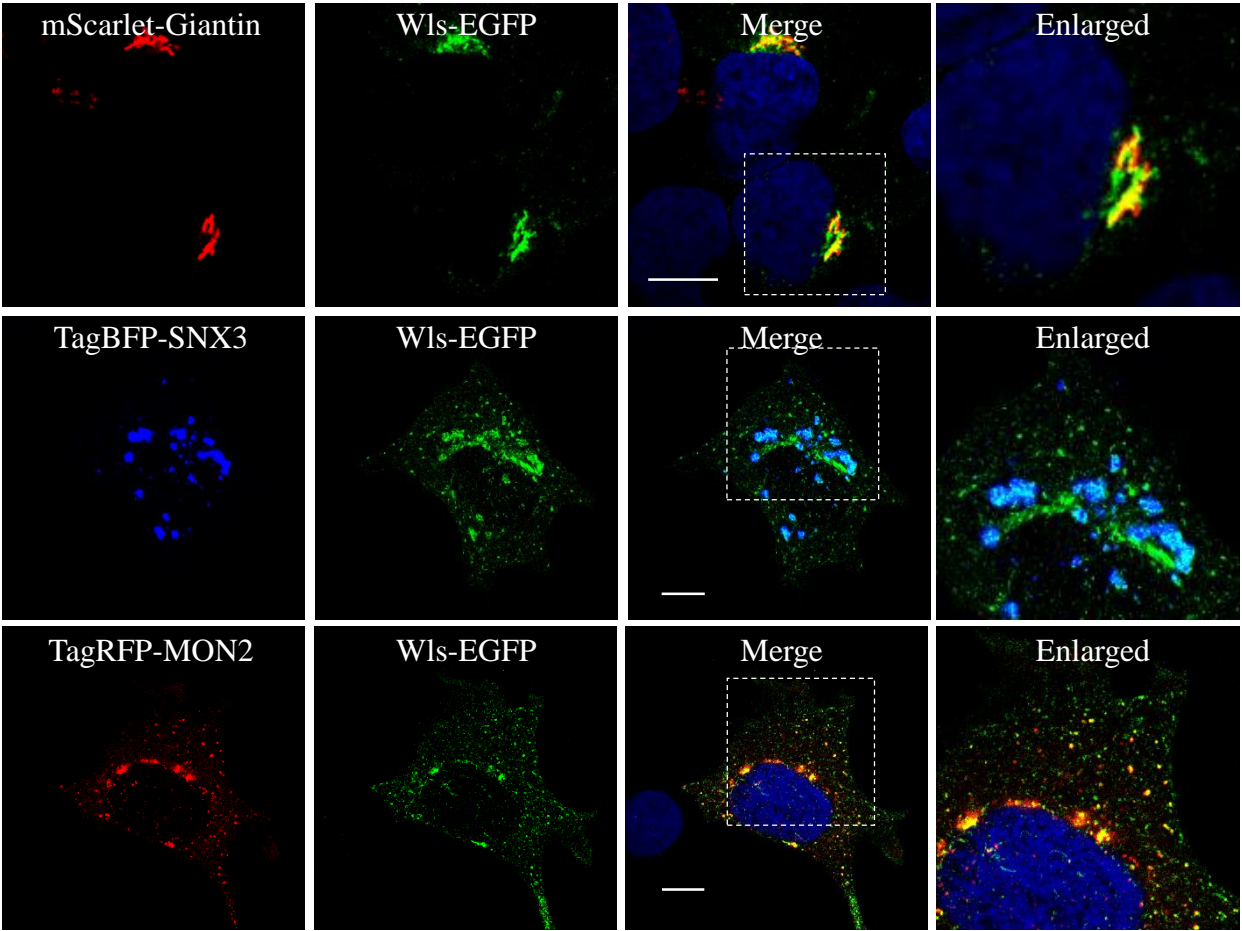

Supplement: Supplementary file 5 — Supplemental Figure S5 [file csf_45_20012_5.pdf]

# Supplemental Figure S6

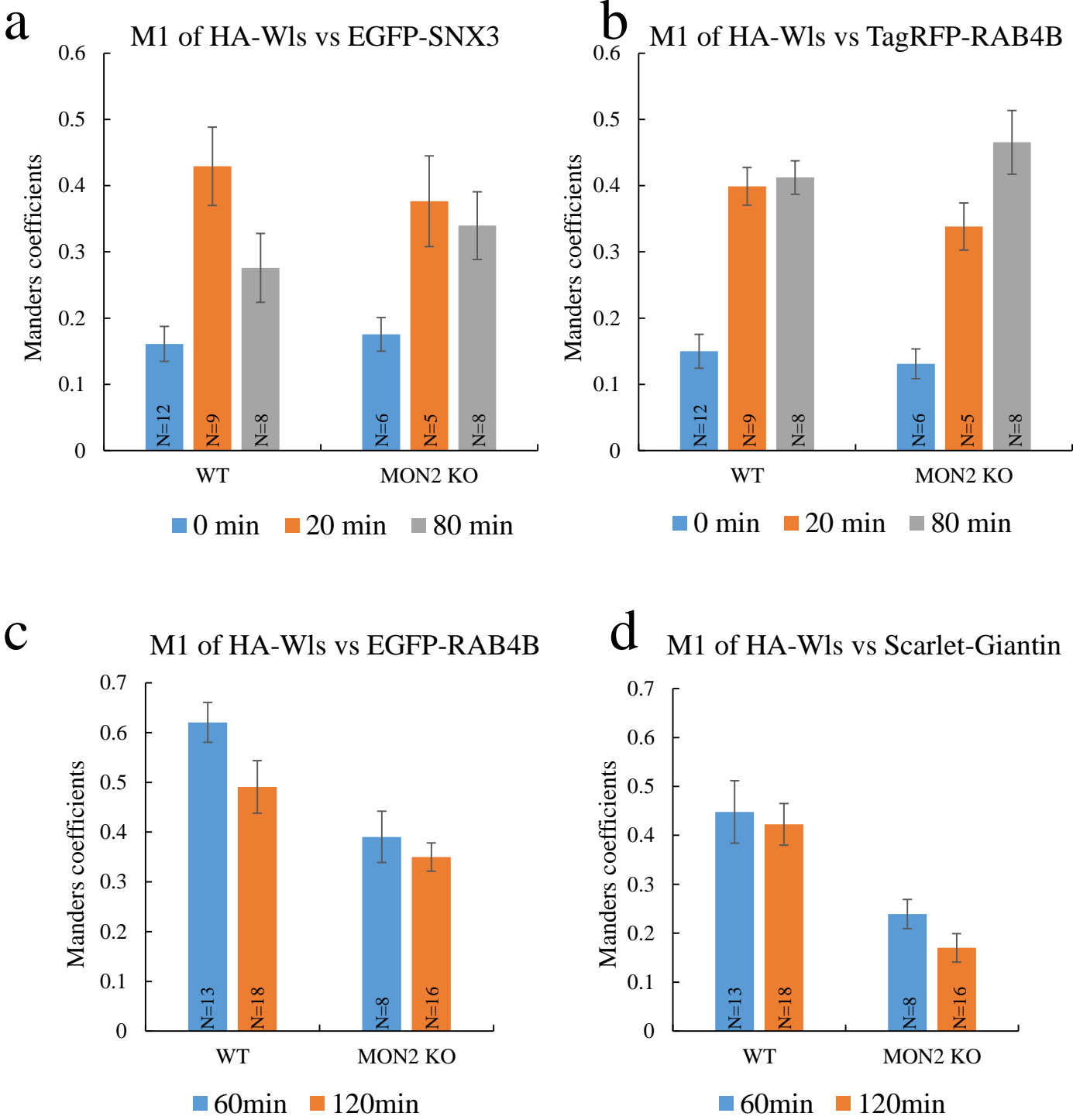

Supplement: Supplementary file 6 — Supplemental Figure S6 [file csf_45_20012_6.pdf]

# Supplemental Figure S7

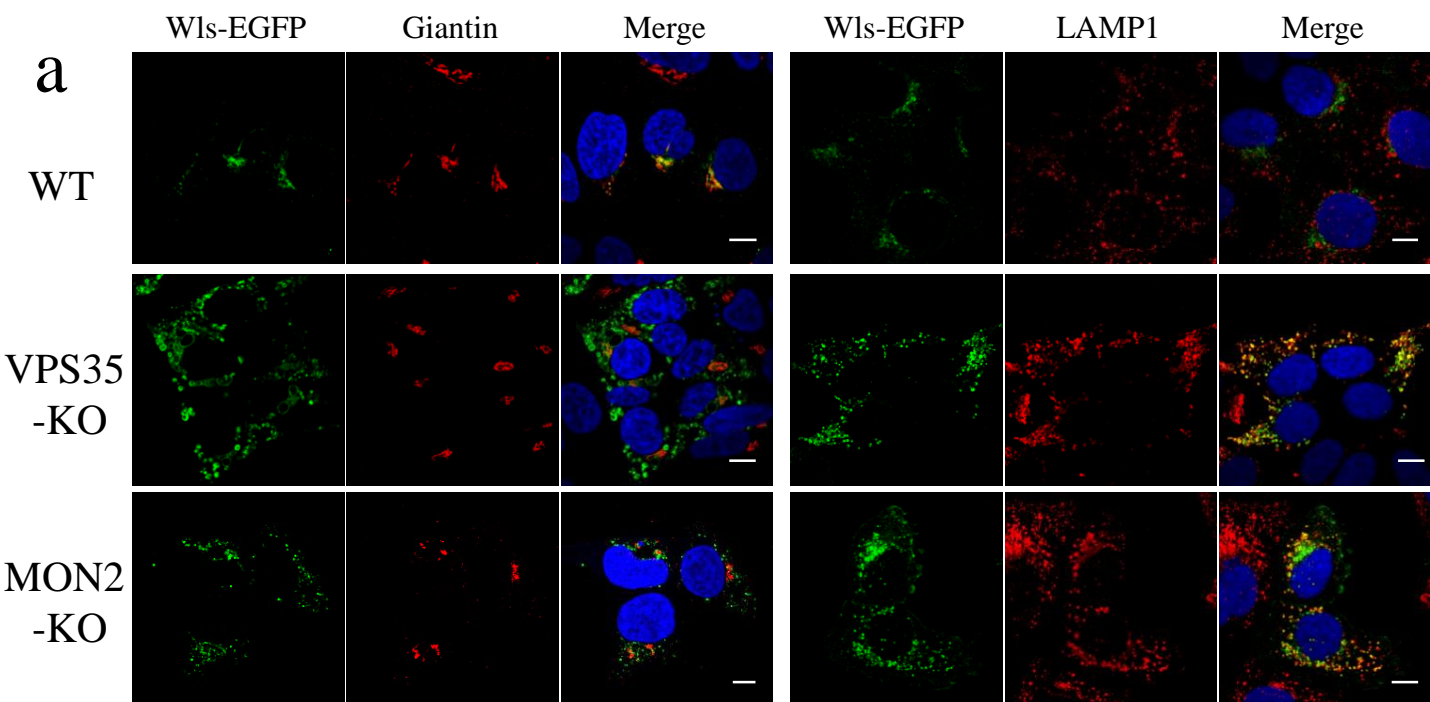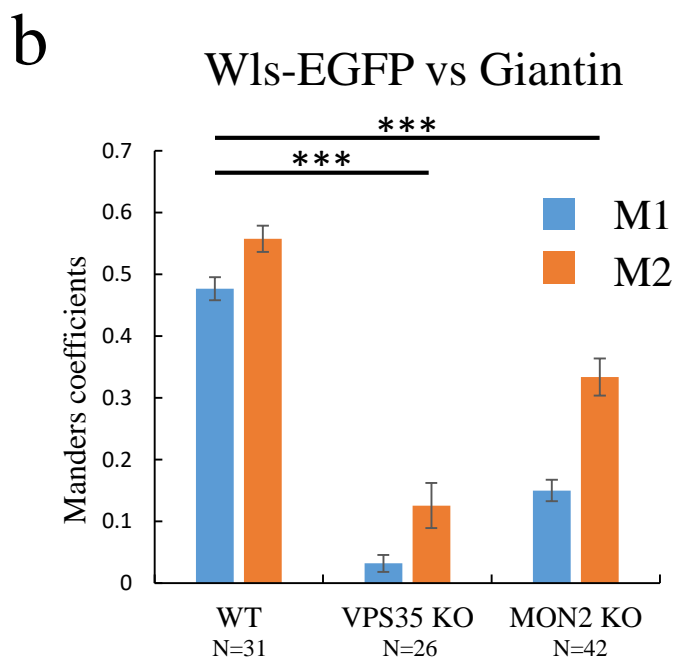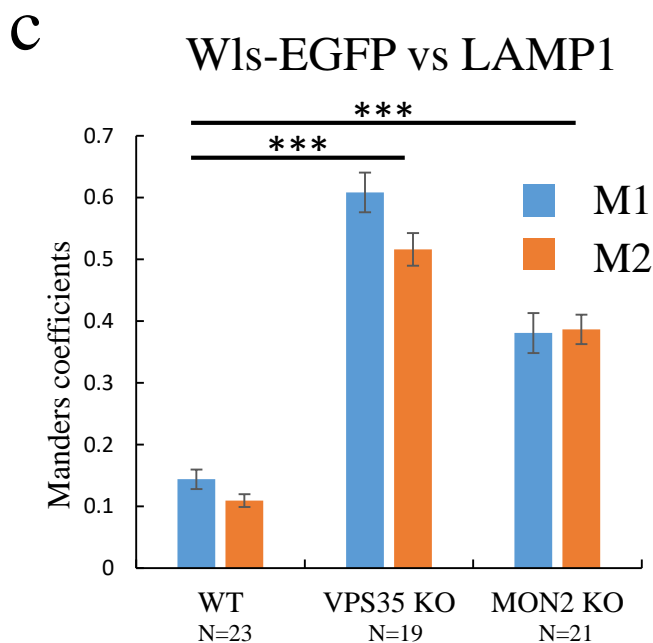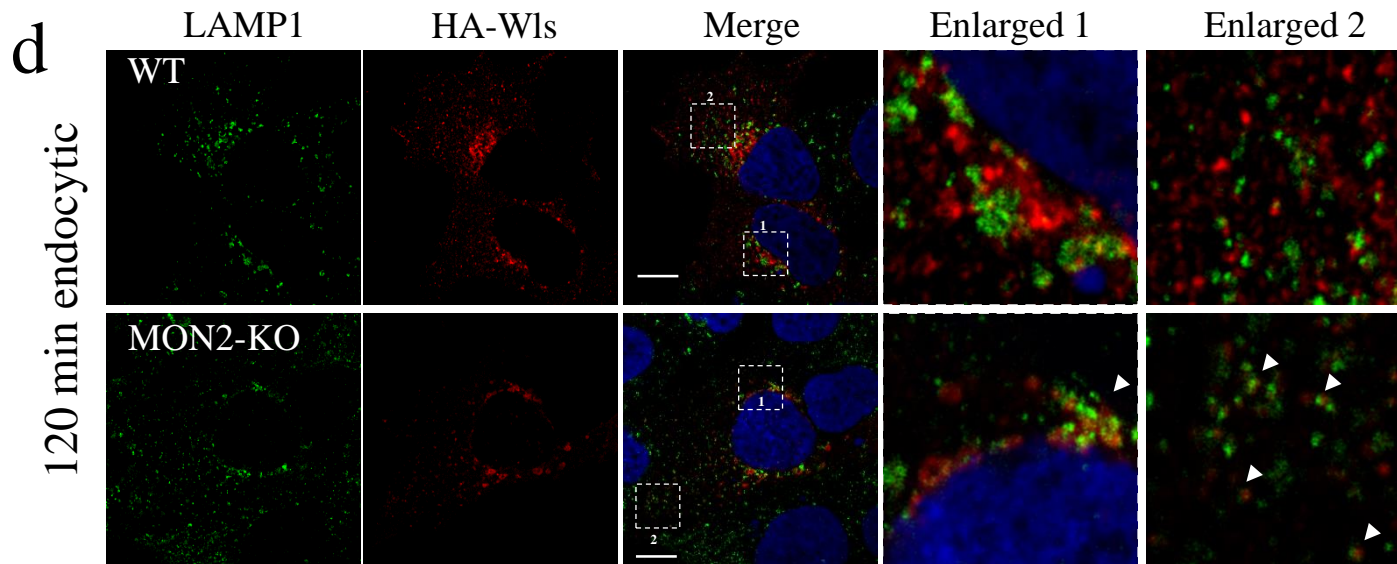

Supplement: Supplementary file 7 — Supplemental Figure S7 [file csf_45_20012_7.pdf]

# Supplemental Figure S8

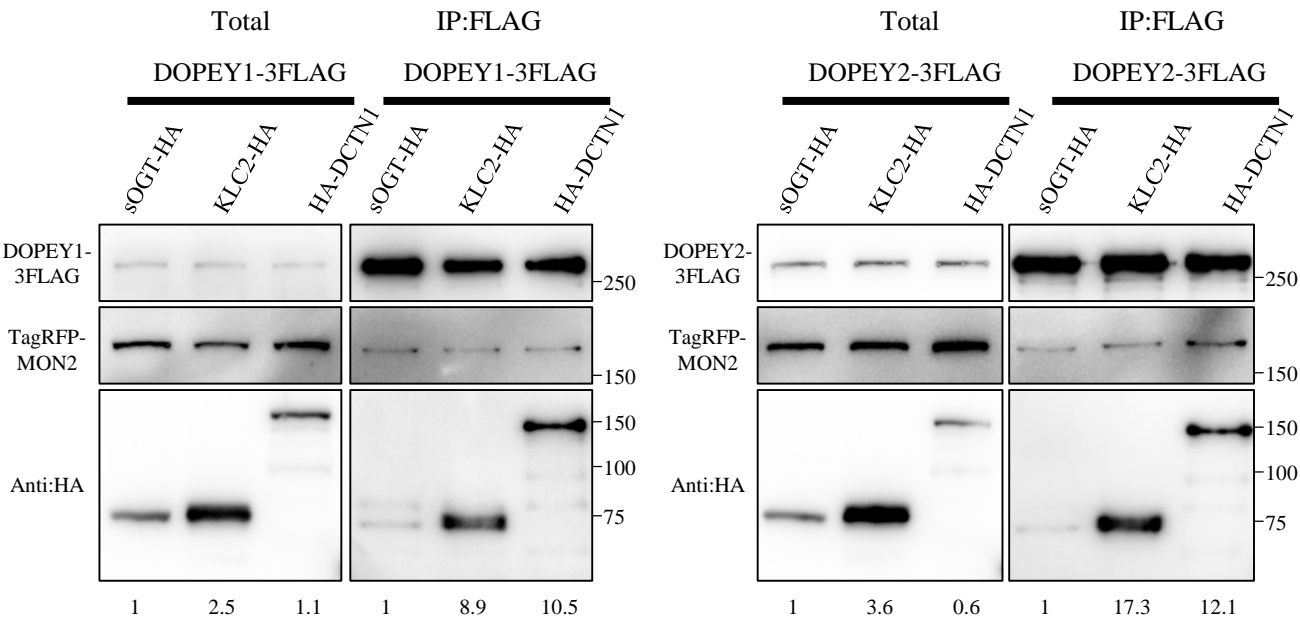

Supplement: Supplementary file 8 — Supplemental Figure S8 [file csf_45_20012_8.pdf]
