# Supplementary material for: MON2 Guides Wntless Transport to the Golgi through Recycling Endosomes
Source: Cell Struct Funct. 2020 May 12;45(1):77–92. doi: 10.1247/csf.20012 (PMC10511057; doi:10.1247/csf.20012)

Supplemental Figure S2

a TagRFP-MON2/mNeonGreen-Giantin/GM130 3h nocodazole

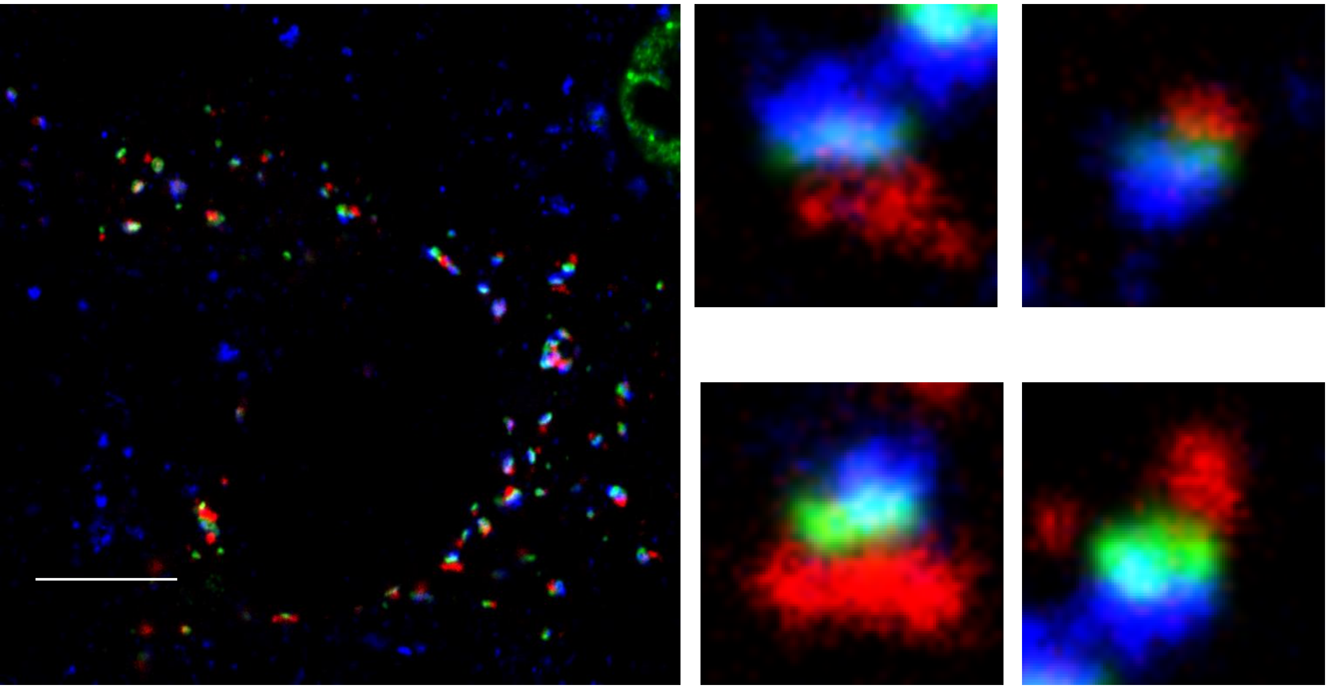

b TagRFP-MON2/mNeonGreen-Giantin/Golgin97 3h nocodazole

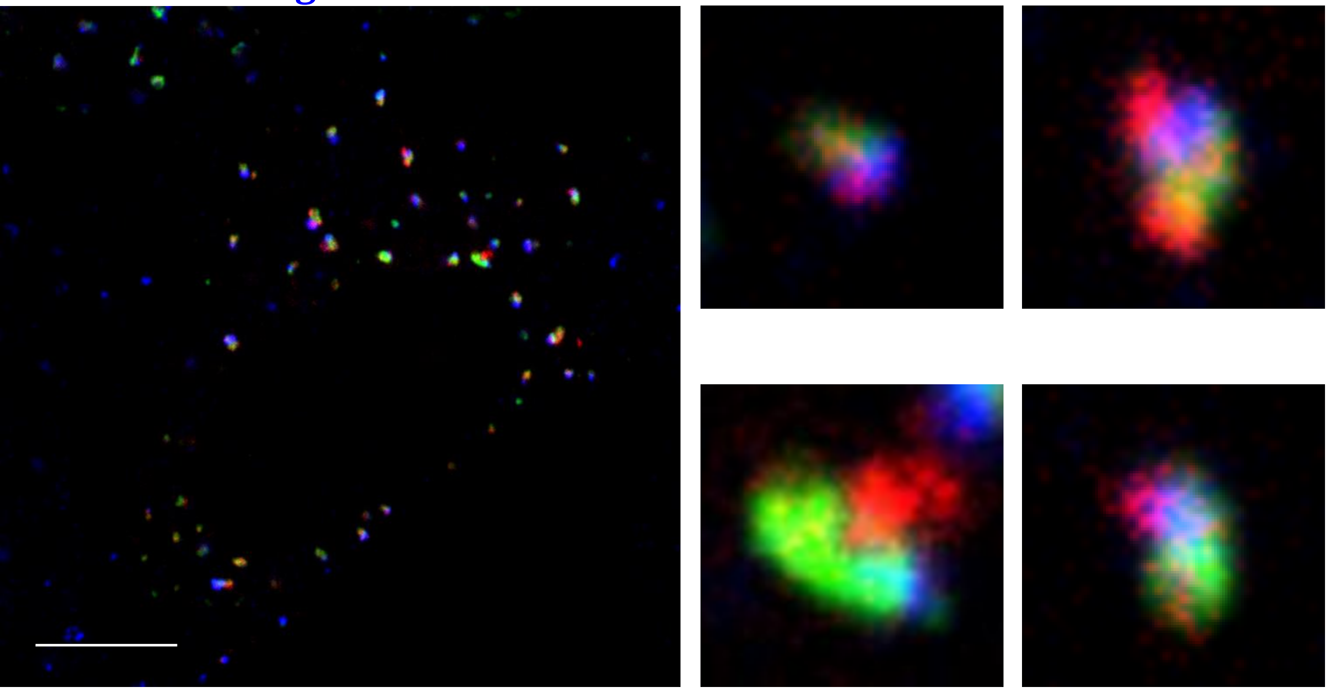

Supplement: Supplementary file 2 — Supplemental Figure S2 [file csf_45_20012_2.pdf]
