# Supplementary material for: MON2 Guides Wntless Transport to the Golgi through Recycling Endosomes
Source: Cell Struct Funct. 2020 May 12;45(1):77–92. doi: 10.1247/csf.20012 (PMC10511057; doi:10.1247/csf.20012)

Supplemental Figure S4

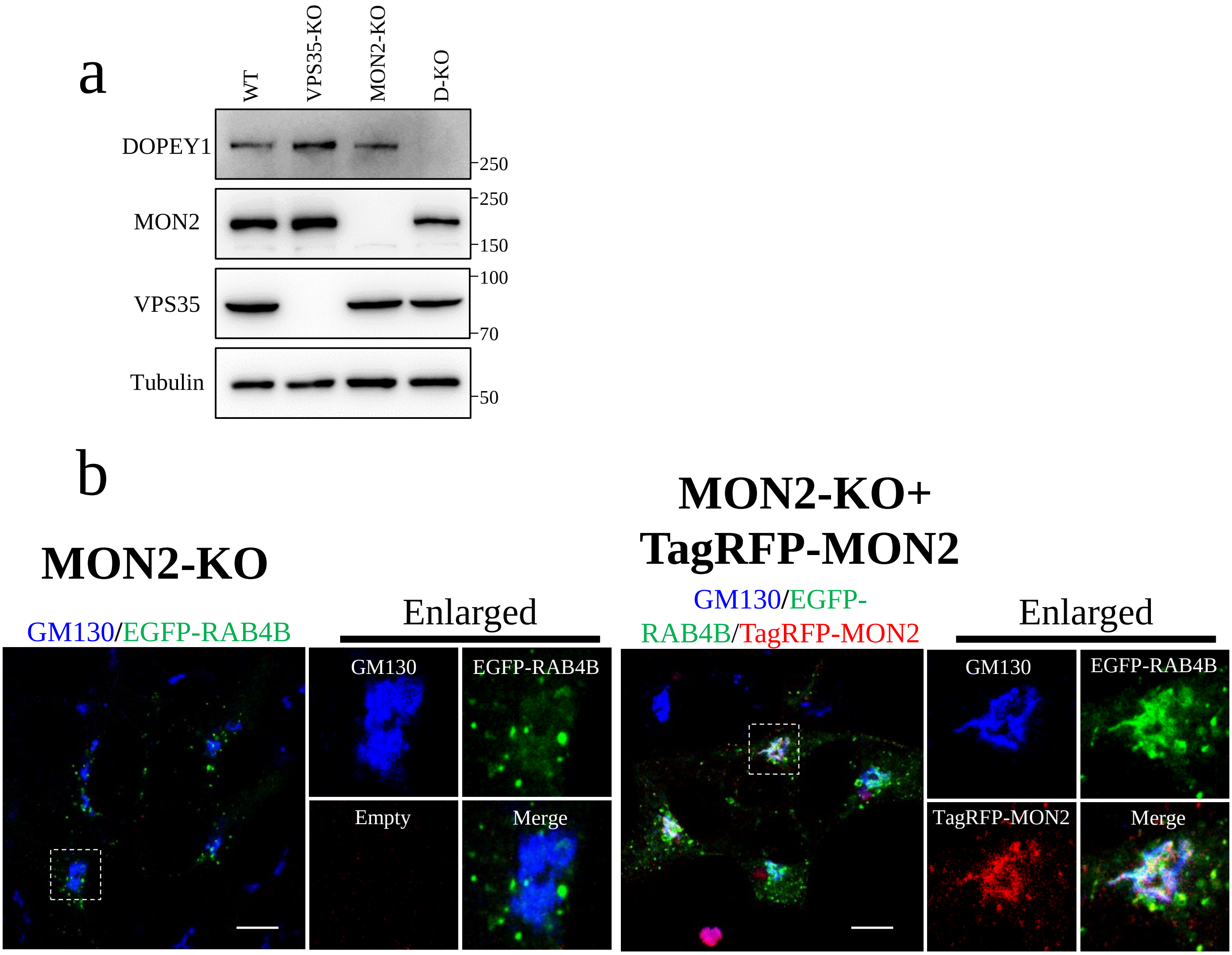

Supplemental Figure S4

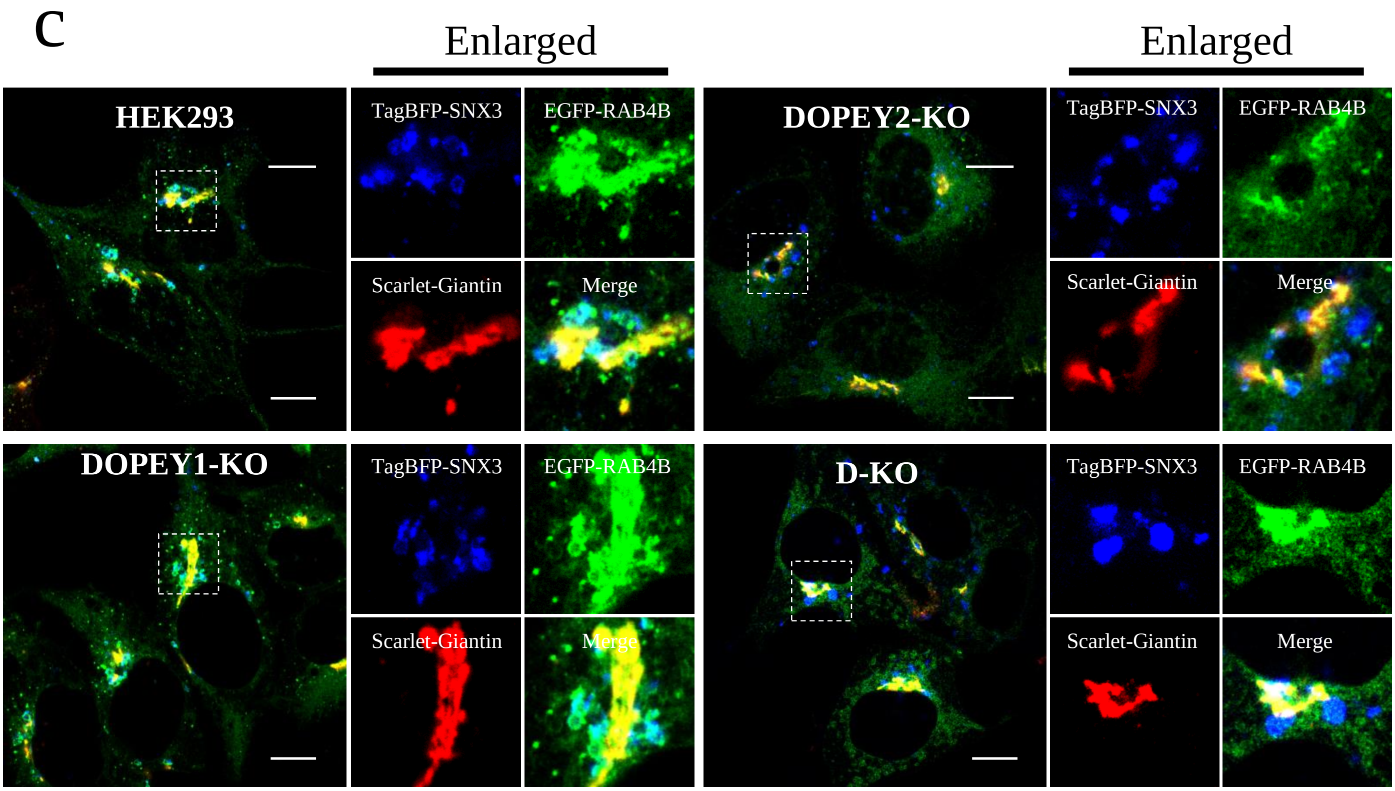

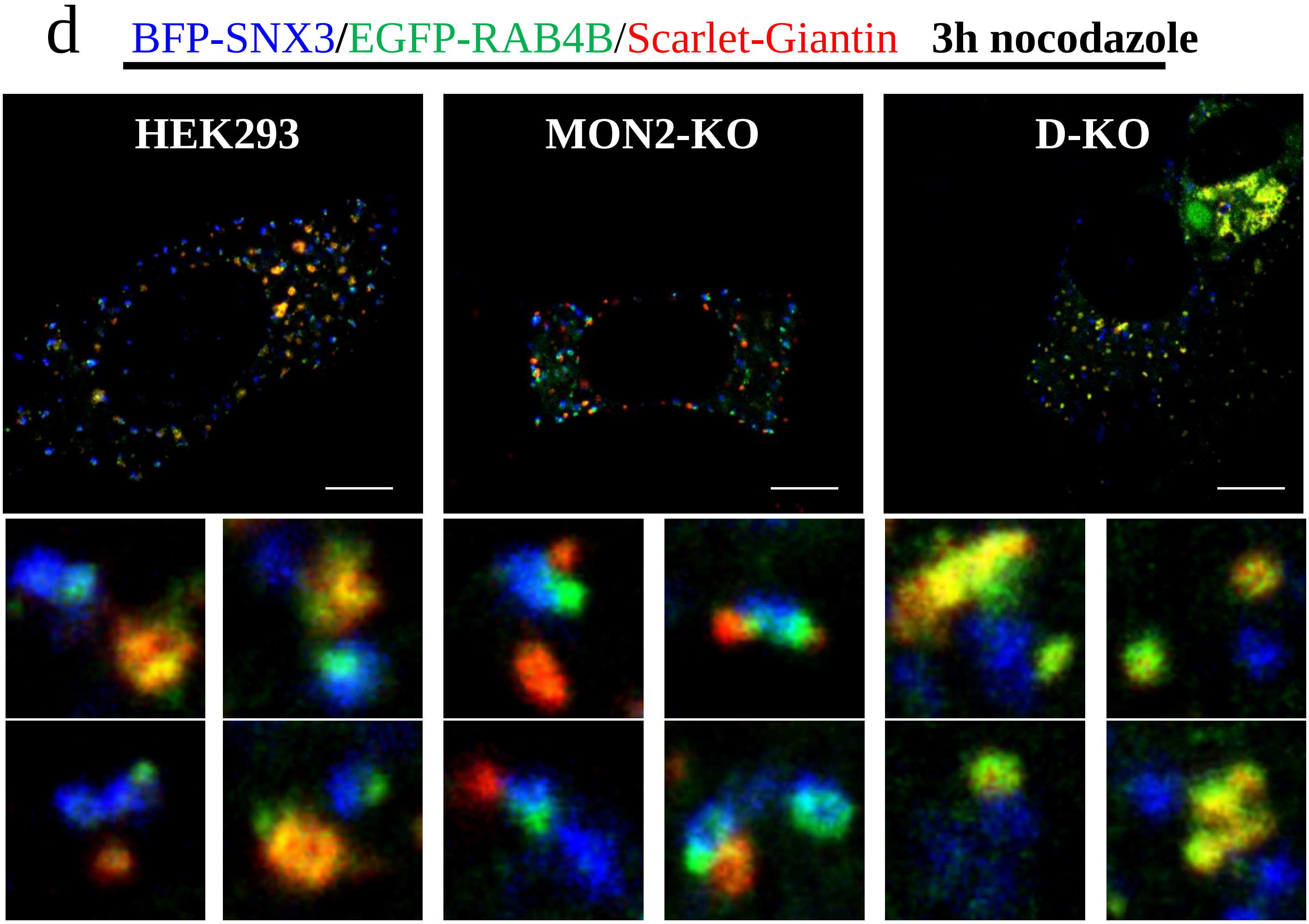

Supplement: Supplementary file 4 — Supplemental Figure S4 [file csf_45_20012_4.pdf]
