# Supplementary material for: MON2 Guides Wntless Transport to the Golgi through Recycling Endosomes
Source: Cell Struct Funct. 2020 May 12;45(1):77–92. doi: 10.1247/csf.20012 (PMC10511057; doi:10.1247/csf.20012)
Supplement: Supplementary file 9 — Supplemental Table S1 [file csf_45_20012_9.pdf]

Supplemental Table S1

```
>MON2-WT
aaaccacagtggctacgagctgttgcggtggaatcaa
>MON2-KO type1(ΔGC)
aaaccacagtggctacgagctgtt..ggtggaatcaa
>MON2-KO type2(+A)
aaaccacagtggctacgagctgttgAcggtggaatcaa

>VPS35-WT
acatcagtgattccatggatttgtactgctcaactttgcagaaatgaacaagctctgggtgcgaatgcagcatcaggg
acatagccgagatagagaaaaaagagaacgagaaagacaagaactgagaattttagtgggaacaaatttggtgcgcctc
agtcagttggaaggtgtaaatgtggaacgtt
>VPS35-KO type1(Δ147bp)
acatcagtgattccatgga............................................................
...............................................................................
........ggaaggtgtaaatgtggaacgtt


>DOPEY1-KO(WT)
AAACGCCTAGCTCAATGTCTACATCCAGCATTACCAGGTGGAGTTCATCGGAAGGCGCTTGAAACATATGAAATTATCT
TCAAAATAATTGGACCTAAGCGACTTGCCAAAGATCTTTTT
>DOPEY1-KO(Δ87bp)
AAACGCCTAGCT...................................................................
....................CGACTTGCCAAAGATCTTTTT


>DOPEY2-KO (WT)
CCTCTCCTGGCACACGCGGCGGTGTCGGTGAGGCCGGTGCTGCTCACCCTGTACGAGAAGTACTTCCTCCCACTGCAGA
>DOPEY2-KO (Δ31bp)
CCTCTCCTGGCACACGCGGCGG...............................CGAGAAGTACTTCCTCCCACTGCAGA


>DOPEY1 in D-KO(WT)
AAACGCCTAGCTCAATGTCTACATCCAGCATTACCAGGTGGAGTTCATCGGAAGGCGCTTGAAACATATGAAATTATCT
TCAAAATAATTGGACCTAAGCGACTTGCCAAAGATCTTTTT
>DOPEY1-KO in D-KO type1(Δ87bp)
AAACGCCTAGCT...................................................................
....................CGACTTGCCAAAGATCTTTTT
>DOPEY1-KO in D-KO type2(Δ88bp)
AAACGCCTAGC....................................................................
....................CGACTTGCCAAAGATCTTTTT


>DOPEY2 in D-KO(WT)
CCTCTCCTGGCACACGCGGCGGTGTCGGTGAGGCCGGTGCTGCTCACCCTGTACGAGAAGTACTTCCTCCCACTGCAGA
>DOPEY2-KO in D-KO (Δ31bp)
CCTCTCCTGGCACACGCGGCGG...............................CGAGAAGTACTTCCTCCCACTGCAGA
```
